# TRIM41 is required to innate antiviral response by polyubiquitinating BCL10 and recruiting NEMO

**DOI:** 10.1038/s41392-021-00477-8

**Published:** 2021-02-28

**Authors:** Zhou Yu, Xuelian Li, Mingjin Yang, Jiaying Huang, Qian Fang, Jianjun Jia, Zheng Li, Yan Gu, Taoyong Chen, Xuetao Cao

**Affiliations:** 1 Center for Systems Medicine, Department of Immunology, Institute of Basic Medical Sciences, Peking Union Medical College, Chinese Academy of Medical Sciences, Beijing, 100005 China; 2 Suzhou Institute of Systems Medicine, Suzhou, 215123 Jiangsu China; 3 National Key Laboratory of Medical Immunology & Institute of Immunology, Second Military Medical University, Shanghai, 200433 China; 4 Institute of Immunology, Zhejiang University School of Medicine, Hangzhou, 310058 China; 5 College of Life Science, Nankai University, Tianjin, 300071 China

**Keywords:** Innate immunity, Inflammation

## Abstract

Sensing of pathogenic nucleic acids by pattern recognition receptors (PRR) not only initiates anti-microbe defense but causes inflammatory and autoimmune diseases. E3 ubiquitin ligase(s) critical in innate response need to be further identified. Here we report that the tripartite motif-containing E3 ubiquitin ligase TRIM41 is required to innate antiviral response through facilitating pathogenic nucleic acids-triggered signaling pathway. TRIM41 deficiency impairs the production of inflammatory cytokines and type I interferons in macrophages after transfection with nucleic acid-mimics and infection with both DNA and RNA viruses. In vivo, TRIM41 deficiency leads to impaired innate response against viruses. Mechanistically, TRIM41 directly interacts with BCL10 (B cell lymphoma 10), a core component of CARD proteins−BCL10 − MALT1 (CBM) complex, and modifies the Lys63-linked polyubiquitylation of BCL10, which, in turn, hubs NEMO for activation of NF-κB and TANK-binding kinase 1 (TBK1) − interferon regulatory factor 3 (IRF3) pathways. Our study suggests that TRIM41 is the potential universal E3 ubiquitin ligase responsible for Lys63 linkage of BCL10 during innate antiviral response, adding new insight into the molecular mechanism for the control of innate antiviral response.

## Introduction

Germline-encoded pattern recognition receptors (PRRs) can recognize pathogen-associated molecular patterns (PAMPs) and host-derived damage-associated molecular patterns (DAMPs), and trigger activation of inflammatory and interferogenic response.^1,2^ Sensing of pathogenic nucleic acids, derived either from pathogens or from host cells, by PRR not only initiates anti-microbe defense but causes inflammatory and autoimmune diseases.^1–3^ More importantly, as pandemic virus spreading has become a worldwide threaten to human health, more works are needed to elucidate the mechanisms involved in innate antiviral response against viral nucleic acids for the sake of control and prevention of virus infection. Ubiquitin (Ub) modification of protein substrates is usually regulated by E3 ubiquitin ligases and plays crucial roles in the control of immunity.^1–3^ Previous studies have identified many E3s for elegant regulation of PRR signaling whereas E3s critical in PRR-associated innate response need to be further identified.

The CARD (caspase recruitment domain) proteins−BCL10 (B cell lymphoma 10)−MALT1 (mucosa-associated-lymphoid tissue lymphoma-translocation gene 1) signalosome, termed as CBM complex, is a kind of multiprotein signaling platform that coordinate diverse receptor signaling pathways to activation of NF-κB in tissue-specific and context-dependent manner.^4^ BCL-10, as the core of CBM complex, contains an amino-terminal CARD that mediates homophilic interactions with the CARDs of CARD9, CARD10 (also known as CARMA3), CARD11 (also known as CARMA1), CARD14 (also known as CARMA2) and BCL-10 itself.^4^ Regarding the control of immune response, the roles of CBM complexes in NF-κB activation downstream various receptors have been well illustrated in T and B cells.^4–6^ Although the CARD-only CARD9 as well as the CARD11-like CARD10 and CARD14 have been coupled to several innate immune receptors,^4,7–10^ thorough understanding of the roles for CBM complex in innate PRR signaling and innate defense has not been achieved. It remains to be determined how the CBM complex is linked to upstream PRR-associated adaptor proteins and downstream NF-κB activation.

It has been revealed that the assembly of CBM complex, especially in lymphocytes, can bridge the downstream signaling pathway by recruiting IκB kinase (IKK) complex and possibly TGFβ-activated kinase 1 (TAK1) complex through NEMO (NF-κB essential modulator, also known as IKKγ), leading to IKKβ-mediated NF-κB activation and MKK6-mediated JNK (c-Jun N-terminal kinase) activation.^4^ NEMO is critical for universal canonical NF-κB activation and for PRR-triggered interferogenic response.^1–3,11–13^ However, the molecular mechanisms bridging the CBM complex with NEMO for canonical NF-κB activation have not been completely elucidated.

Within the CBM complex, BCL10 acts as a scaffold for CBM assembly and is important in activation of IKK and NF-κB.^4–6,14^ Previous studies have suggested that posttranslational modifications of BCL10 may be the potential mechanisms for regulation of IKK activity.^4^ BCL10 is phosphorylated in response to TCR (T cell receptor) activation, which has been reported to regulate IKK and NF-κB activation.^15–18^ BCL10 also undergoes ubiquitination in response to TCR ligation, which has been thought to target BCL10 for degradation and thus to down-regulate NF-κB activation.^19,20^ Although Lys63 linkage of BCL10 is required for NF-κB activation upon TCR ligation,^21^ the universal E3 ubiquitin ligase(s) mediating Lys63-linkage of BCL10 during innate immune signaling, have not been identified.

Our group has identified several key E3 ubiquitin ligases involved in the regulation of immune response.^22–25^ We provide genetic and in vivo evidence here for the critical roles of TRIM41, one of the tripartite motif (TRIM) family proteins, in innate antiviral response. More importantly, we identify TRIM41-mediated interaction with and Lys63-linked polyubiquitination of BCL10 as the missing hub for NEMO recruitment, casting new light on the mechanistic understanding of CBM complex in prevalent control of innate antiviral response.

## Results

### TRIM41 is required for pathogenic nucleic acid- and virus-triggered innate response

Previously we have screened the gene expression profile in RAW264.7 macrophages after infection with the RNA virus vesicular stomatitis virus (VSV) and the DNA virus herpes simplex virus 1 (HSV-1).^25^ Among the *Trim* genes, we noted that *Trim41* mRNA was downregulated by virus infection (GEO accession number GSE72077). After searching the gene databases GeneCards and BioGPS, we realized that *Trim41* was ubiquitously expressed in human and mouse tissues, and was highly expressed by immune cells including T cells, B cells, NK cells, dendritic cells, and macrophages (bone marrow-derived macrophages−BMDM, peritoneal macrophages, and RAW264.7 cells). These data suggest that *Trim41* may play a role in immune response.

TRIM41 has been identified as PKC isozymes-interacting E3 ubiquitin ligase and as inhibitor of virus replication.^26–28^ However, its role in innate immune response has not been characterized at the stage of conceptualization and design of the study. So we examined the roles of *Trim41* in innate antiviral response. First, we found that *Trim41* was significantly downregulated in peritoneal macrophages after VSV treatments (Supplementary Fig. S1a, b). Second, we found that *Trim41* knockdown (KD) (Supplementary Fig. S1c) inhibited the mRNA expression of *Il6* and *Ifnb* induced by VSV (Supplementary Fig. S1d, e). The production of IL-6 and IFNβ induced by VSV was also significantly inhibited after *Trim41* knockdown (Supplementary Fig. S1f, g). After infection with HSV-1, *Trim41* mRNA and protein were also downregulated (Supplementary Fig. S1a, b). *Trim41* knockdown also significantly inhibited the production of IL-6 and IFNβ induced by HSV-1 (Supplementary Fig. S1f, g). So TRIM41 may be positively involved in innate antiviral response. Previously several TRIM molecules have been implicated in the positive regulation of innate antiviral response.^29–32^ The mRNA expression of *Trim25*, *Trim31*, *Trim32* or *Trim56* in peritoneal macrophages could be modulated by VSV and HSV-1 infections (Supplementary Fig. S2a-d). After knockdown of *Trim25*, *Trim31*, *Trim32* or *Trim56* (Supplementary Fig. S2e, f), we found that the production of IL-6 and IFNβ induced by VSV or HSV-1 infection was significantly inhibited (Supplementary Fig. S2g-j), resembling to the effects of *Trim41* knockdown. These preliminary data provoked us to investigate the roles of *Trim41* in the innate antiviral response.

**Fig. 1:**
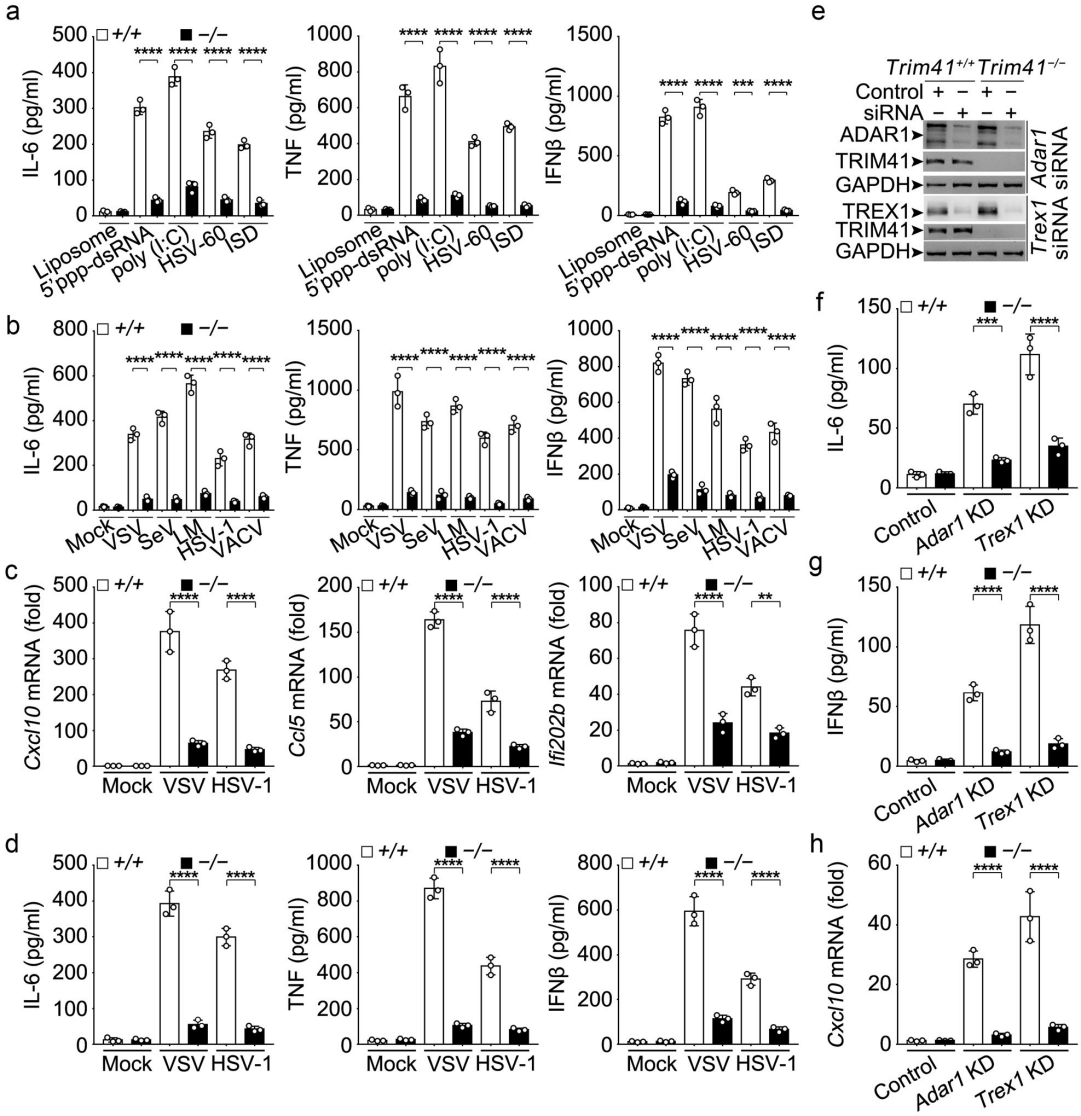
TRIM41 deficiency impairs innate response to pathogenic nucleic acid mimics and virus infections. **a**–**d** *Trim41*^*+/+*^ or *Trim41*^*–/–*^ BMDM (1×10^5^ cells per 24-well; **a**–**c**) or BMDC (1 × 10^5^ cells per 24-well; **d**) were treated with liposome-packaged synthesized cytosolic nucleic acids sensor agonists for 4 h (**a**; 500 ng/ml 5′ppp-dsRNA for RIG-I, 500 ng/ml poly (I:C) for MDA5, 2 μg/ml HSV-60 or ISD for cGAS−STING), or indicated pathogens for 8 h (**b**–**d**) (MOI = 1 for VSV and SeV, MOI = 2 for LM, and MOI = 5 for HSV-1 or VACV). Amounts of IL-6, TNF, and IFNβ in supernatants were measured by ELISA (**a**, **b** and **d**), and the mRNA levels of ISGs were examined by Q-PCR (**c**). **e** 48 h after the transfection of *Trim41*^*+/+*^ or *Trim41*^*–/–*^ BMDM cells with control or *Trex1*- or *Adar1*-specific siRNAs, the expression of indicated proteins were examined by Western blotting. **f-h** Cells in (**e**) were cultured in fresh medium for 48 h. Amounts of IL-6 and IFNβ in the supernatants were measured by ELISA (**f** and **g**), and the mRNA levels of *Cxcl10* were examined by Q-PCR (**h**). Results are presented as mean ± SD of three biological replicates (**a**–**d** and **f**–**h**; one-way ANOVA followed by Bonferroni multiple comparison). One representative experiment of three is shown. **P < 0.01; ***P < 0.001; ****P < 0.0001

Next we established *Trim41* knockout mice (Supplementary Fig. S3) to further investigate the role of TRIM41 in innate antiviral response in BMDM. We first examined the roles of TRIM41 in cytosolic nucleic acid-induced innate response by using liposome-packaged cytosolic agonistic nucleic acids. We found that the production of IL-6, TNF, and IFNβ in *Trim41*^*−/−*^ BMDM was significantly reduced after treatments with either cytosolic RNA sensor agonists (5′ppp-dsRNA for RIG-I, and poly (I:C) for MDA-5) or cytosolic DNA sensor agonists (60 bp oligonucleotide containing HSV-1 DNA motifs–HSV60 and interferon stimulatory DNA–ISD) (Fig. 1a). Furthermore, when *Trim41*^*−/−*^ BMDM were infected with RNA viruses (VSV and Sendai virus–SeV), intracellular bacteria *Listeria monocytogenes* (LM), and DNA viruses (HSV-1 and Vaccina virus–VACV), we found that these pathogens-induced production of IL-6, TNF, and IFNβ cytokines was also significantly reduced (Fig. 1b). As further evidence for TRIM41-mediated virus-triggered interferogenic response, we found that TRIM41 deficiency impaired the induction of interferon-stimulated genes (ISG), such as *Cxcl10*, *Ccl5*, and *Ifi202b*, by virus infections (Fig. 1c). In bone marrow-derived dendritic cells (BMDC), we also found that TRIM41 was critical in virus-triggered innate response (Fig. 1d). Taking together the data for cytosolic agonists and pathogens, we propose that TRIM41 is required for inflammatory and interferogenic innate response triggered by pathogenic nucleic acid mimics and viruses.

ADAR1 (adenosine deaminase acting on RNA 1) catalyzes the hydrolytic deamination of adenosine to inosine in host dsRNA, and TREX1 (three prime repair exonuclease 1) catalyzes host DNA degradation, both of which have been implicated in preventing innate response to pathogenic self-nucleic acids.^3,33,34^ After knockdown (KD) of *Adar1* and *Trex1* in *Trim41*^*+/+*^ and *Trim41*^*−/−*^ BMDM (Fig. 1e), we found that *Trim41*^*+/+*^ BMDM autonomously produced IL-6 and IFNβ and upregulated *Cxcl10* expression while TRIM41 deficiency reversed this response (Fig. 1f–h). Therefore TRIM41 is also critical in innate responses to pathogenic self-nucleic acids.

### TRIM41 deficiency impairs innate antiviral defense in vivo

**Fig. 2:**
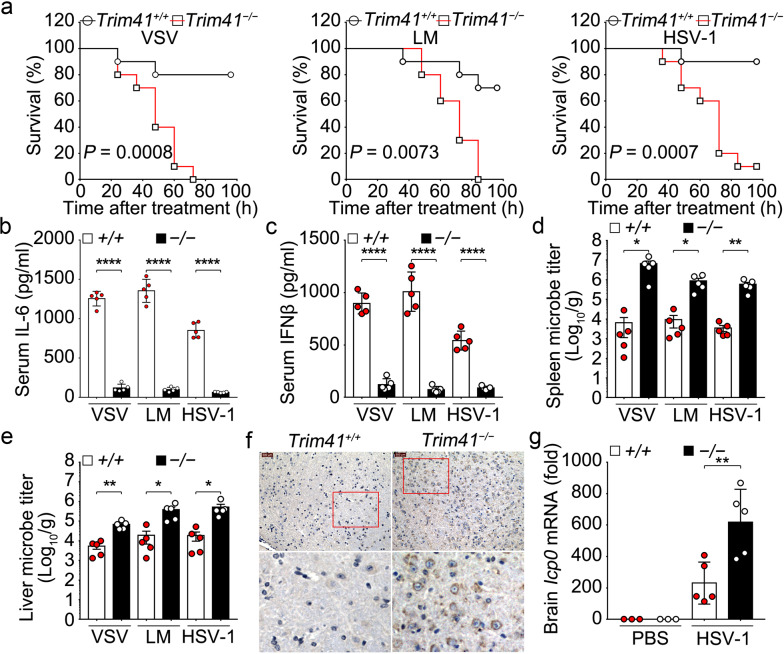
TRIM41 deficiency impairs innate response and exaggerates virus propagation in vivo. **a-e** *Trim41*^*+/+*^ or *Trim41*^*–/–*^ mice were intraperitoneally injected with 100 μl of PBS or PBS containing 5 × 10^6^ PFUs of VSV or LM, or 2 × 10^8^ PFUs of HSV-1 as indicated. The survival of mice (*n* = 10 per group) was monitored by Kaplan and Meier method and analyzed by Log-rank test (**a**). Otherwise, 12 h after pathogen infections, serum levels of IL-6 (**b**) and IFNβ (**c**) was measured by ELISA (*n* = 5 mice per group). Alternatively, 24 h after pathogen infections, the microbe titers in the spleen and liver (three biological samples derived from each mouse; *n* = 5 per group) were determined by plaque formation assays (**d**, **e**). **f**, **g** *Trim41*^*+/+*^ or *Trim41*^*–/–*^ mice were intravenously injected with 100 μl of PBS or PBS containing 2 × 10^8^ PFUs of HSV-1 as indicated. 72 h after treatments, the replication status of HSV-1 in the brain was evaluated by IHC staining of HSV1 (representative images are shown; **f**) or by Q-PCR assays of *Icp0* mRNA (three biological samples derived from each mouse; *n* = 3 mice for the PBS group, and *n* = 5 mice for the HSV-1 group; **g**). Results are presented as mean ± SE (**b**–**e** and **g**, one-way ANOVA followed by Bonferroni multiple comparison). One representative experiment of three is shown. One representative experiment of three is shown. *P < 0.05; **P < 0.01; ****P < 0.0001

Since the innate response to pathogenic nucleic acids and virus infection has been impaired in *Trim41*^*−/−*^ BMDM and BMDC, we further focused on investigation of TRIM41-mediated innate antiviral defense by using VSV, *Listeria monocytogenes* (LM) and HSV-1 infection models. After intraperitoneal injection of these microbes, we found that the survival of *Trim41*^*−/−*^ mice was significantly shortened (Fig. 2a) despite that PBS treatments didn’t affect the survival of either *Trim41*^*+/+*^ or *Trim41*^*−/−*^ mice. The serum levels of IL-6 (Fig. 2b) and IFNβ (Fig. 2c) were significantly decreased in *Trim41*^*−/−*^ mice, and the microbe titers were significantly increased in the spleen and liver of *Trim41*^*−/−*^ mice (Fig. 2d, e). When HSV-1 viruses were intravenously injected, we found that the replication of HSV-1 in the brain was significantly enhanced in *Trim41*^*−/−*^ mice (Fig. 2f, g). These data suggest that TRIM41 is required for the protection of the host from virus invasion.

### TRIM41 potentiates virus-triggered NF-κB and IRF3 activation

**Fig. 3:**
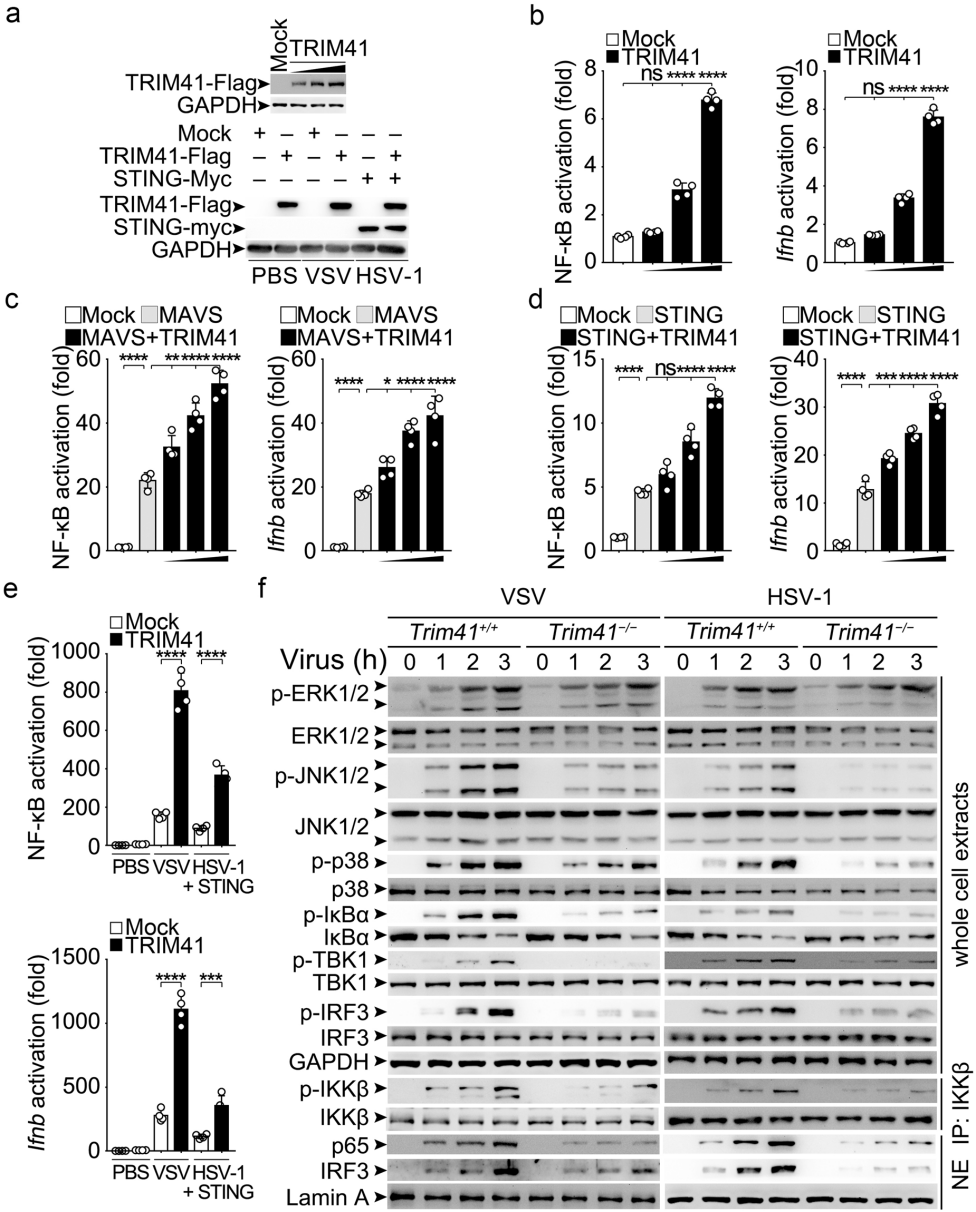
TRIM41 potentiates the activation of both NF-κB and IRF3 during virus infection. **a**–**e** HEK293T cells were cotransfected with mock or Flag-tagged TRIM41 (10, 50, or 100 ng) vectors, vectors for PRR adaptors (100 ng MAVS or STING), and reporter vectors. 48 h later, the expression of TRIM41-Flag and STING-Myc was examined by Western blotting (**a**). Then the cells were infected with (**e**) or without (**b**–**d**) VSV (MOI = 1) or HSV-1 (MOI = 5) for 4 h, and the luciferase activity was determined (**b-e**). **f** *Trim41*^*+/+*^ or *Trim41*^*–/–*^ BMDM were infected with VSV (MOI = 1) or HSV-1 (MOI = 5) viruses as indicated. The activation of the signaling mediators were examined by Western blotting. Results (**b**–**e**) are presented as mean ± SD of four biological samples (one-way ANOVA followed by Bonferroni multiple comparison). One representative experiment of three is shown. ns, not significant; *P < 0.05; ***P < 0.001; ****P < 0.0001

How does TRIM41 regulate the pathogenic nucleic acids sensing signaling pathways and thus contribute to innate antiviral response? To answer this question, we examined the signaling pathways by using gene reporters. We found that TRIM41 overexpression (Fig. 3a, upper panel) dose-dependently activate NF-κB and *Ifnb* reporters (Fig. 3b). When co-transfected with the core adaptors of cytosolic nucleic acid sensing pathways (mitochondria antiviral signaling protein–MAVS as RNA sensing adaptor and stimulator of interferon genes–STING as DNA sensing adaptor),^1–3^ TRIM41 could dose-dependently potentiate MAVS- and STING- (Fig. 3c, d) induced activation of NF-κB and *Ifnb* reporters. However, TRIM41 overexpression could not enhance p65 (also known as RelA)-induced activation of NF-κB reporters and interferon regulatory factor 3 (IRF3)-induced activation of *Ifnb* reporters (Supplementary Fig. S4a, b). When HEK293T cells were infected with VSV or HSV-1 (after transfection of STING-Myc vectors), TRIM41 overexpression (Fig. 3a, lower panel) still enhance the activation of NF-κB and *Ifnb* reporters (Fig. 3e). These data suggest that TRIM41 may act downstream of MAVS and STING but upstream of transcription factors.

To explore the effects of TRIM41 on innate antiviral signaling pathway in detail, we examined the MAPK (mitogen-activated protein kinase) pathway (including ERK1/2, JNK1/2, and p38), the IKKβ − IκBα − p65 pathway, and the TBK1 − IRF3 pathway in *Trim41*^*−/−*^ BMDM after treatments with VSV and HSV-1. We found that TRIM41 deficiency inhibited JNK1/2 and p38 (but not ERK1/2) activation, decreased the phosphorylation of IKKβ, IκBα, TBK1, and IRF3, and delayed the nuclear translocation of p65 and IRF3 (Fig. 3f). These data firmly support the proposal that TRIM41 is required for virus-triggered NF-κB and IRF3 activation.

### TRIM41 complexes with CARD proteins and directly interacts with BCL10

**Fig. 4:**
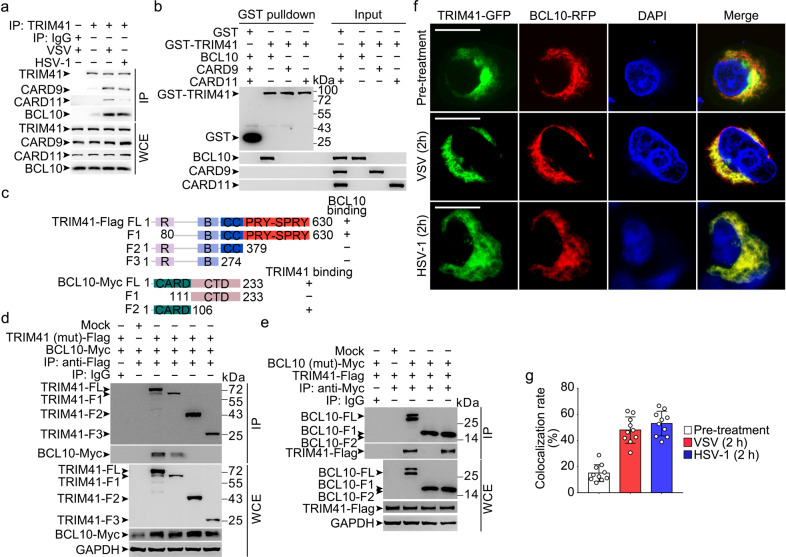
The PRY-SPRY domain of TRIM41 directly binds the CARD domain of BCL10. **a** Wild type BMDM were infected with VSV (MOI = 1) or HSV-1 (MOI = 5) viruses for 2 h as indicated. Then whole cell extracts (WCE) were immunoprecipitated with anti-TRIM41 antibody plus protein A/G beads. Components in the TRIM41 complex were examined by Western blotting. **b** 1 μg GST or GST-TRIM41 was coincubated with 1 μg recombinant CARD9, CARD11, or BCL10 for 1 h. Then sepharose 4B beads were used to pull down the GST complex. Components pulled down by the beads were examined by Western blotting. **c** Schematic illustration of domains in full-length (FL) or fragments (F) of TRIM41-Flag or BCL10-Myc. R, ring finger domain; B, B-box domain; CC, coiled-coil domain; CTD, C-terminal domain. Numerical numbers indicate the site of amino acids. **d**, **e** HEK293T cells were transiently transfected with Flag-tagged TRIM41 (or mutants) and Myc-tagged BCL10 (or mutants) as indicated for 48 h. Whole cell extracts (WCE) were immunoprecipitated with anti-Flag Sepharose Beads (**d**) or anti-Myc Sepharose Beads (**e**), and the associated BCL10 or TRIM41 was examined by Western blotting. **f**, **g** RAW264.7 cells grown on cover slides were transiently transfected with green fluorescent protein (GFP)-tagged TRIM41 and red fluorescent protein (RFP)-tagged BCL10 for 48 h, and then infected with VSV (MOI = 1) or HSV-1 (MOI = 5) viruses for 2 h. After counterstained with DAPI, cells were examined by confocal microscope (**f**; Scale bar, 100 nm). Ten double-positive randomly-selected cells on cover slides were measured for fluorescence colocalization, and the data are presented as mean ± SD (**g**)

TRIM41 is an E3 ubiquitin ligase candidate,^35^ and E3 ubiquitin ligases usually provide the specificity for signaling control via interaction with and Ub-modification of protein substrates.^1–3^ So we went to identify TRIM41 partners by using immunoprecipitations (IP) after overexpression of Flag-tagged TRIM41 in HEK293T cells, followed by mass spectrometry. Among the identified proteins interacting with TRIM41 (Supplementary Table S1), we noted the CARD14, one component of the CBM complex and seldom identified in E3 ubiquitin ligase complexes. So we examined the interaction of endogenous TRIM41 with CARD proteins and BCL10 in BMDM. We found that TRIM41 could bind with BCL10 as well as CARD9 and CARD11, which was enhanced by VSV and HSV-1 treatments (Fig. 4a). By using GST (glutathione S-transferase)-fusion TRIM41 as well as recombinant BCL10, CARD9, or CARD11 in GST pull-down assays, we found that TRIM41 could directly bind BCL10, but not CARD9 and CARD11 (Fig. 4b).

After cotransfection of Flag-Tagged TRIM41 (mutants) with Myc-tagged BCL10 (mutants) as illustrated in Fig. 4c, we found that the PRY-SPRY domain in TRIM41 could mediate the interaction with the CARD domain of BCL10 (Fig. 4d, e). Furthermore, we found that TRIM41 was partially colocalized with BCL10 in cytoplasm after overexpression in RAW264.7 macrophages while VSV and HSV-1 treatments could enhance the colocalization (Fig. 4f, g). Therefore, BCL10, and possibly TRIM41-associated CARD9 and CARD11, may be the substrate for TRIM41 modification during innate response.

### TRIM41 acts as the E3 ubiquitin ligase mediating Lys63-linked polyubiquitination of BCL10

**Fig. 5:**
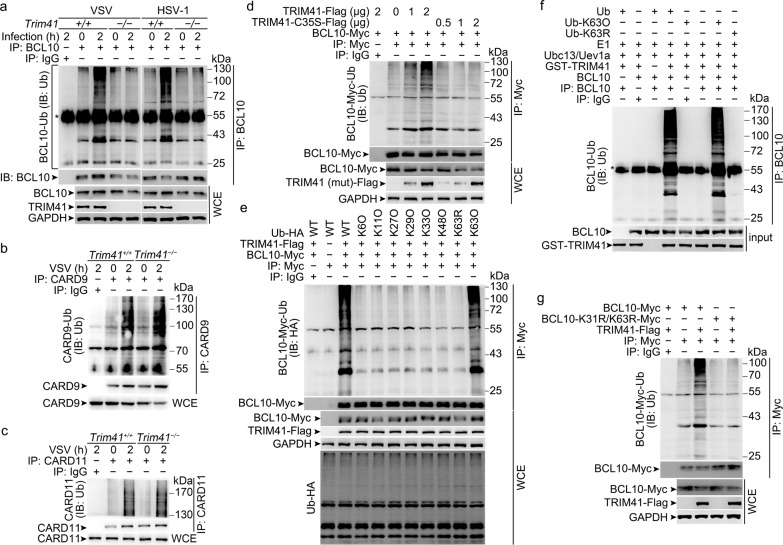
TRIM41 mediates Lys63-linked polyubiquitination of BCL10. **a**-**c** *Trim41*^*+/+*^ or *Trim41*^*–/–*^ BMDM were infected with VSV (MOI = 1) or HSV-1 (MOI = 5) viruses for 2 h. Then whole-cell extracts (WCE) heated in buffer containing 1% SDS were immunoprecipitated (IP) with anti-BCL10 (**a**), anti-CARD9 (**b**), or anti-CARD11 (**c**) antibody plus protein A/G beads. Polyubiquitination of BCL10 (**a**), CARD9 (**b**), and CARD11 (**c**) was examined by Western blotting. **d** HEK293T cells were transiently transfected with Flag-tagged TRIM41 (or mutant) and Myc-tagged BCL10 vectors as indicated for 48 h. Then polyubiquitination of BCL10 was examined by Western blotting after immunoprecipitations with anti-Myc Sepharose Beads. **e** HEK293T cells were transiently transfected with Flag-tagged TRIM41, Myc-tagged BCL10, and HA-tagged Ub (mutants) vectors as indicated for 48 h. Then polyubiquitination of BCL10 was examined by Western blotting after immunoprecipitations with anti-Myc Sepharose Beads. **f** After incubation for 30 min, the in vitro polyubiquitination system was boiled for 5 min, and then polyubiquitinated BCL10 was examined by Western blotting against Ub after immunoprecipitations. **g** HEK293T cells were transiently transfected with Flag-tagged TRIM41 and Myc-tagged BCL10 (mutant) vectors as indicated for 48 h. Then polyubiquitination of BCL10 was examined by Western blotting after immunoprecipitations with anti-Myc Sepharose Beads. One representative experiment of three is shown

As an E3 ubiquitin ligase, TRIM41 may promote the ubiquitylation of the substrate(s). As expected, we found that polyubiquitination of BCL10 was decreased in *Trim41*^*−/−*^ BMDM after VSV and HSV-1 treatments (Fig. 5a) while the ubiquitylation of CARD9 and CARD11 was not remarkably affected (Fig. 5b, c). In HEK293T cells, TRIM41 could dose-dependently enhance BCL10 polyubiquitination, which required the E3 ligase activity of TRIM41 since mutation of Cys35 into Ser (TRIM41-C35S, enzyme activity deficiency) in the ring finger domain could not promote BCL10 polyubiquitination (Fig. 5d).

To identify the Ub-modification type of BCL10 by TRIM41, we cotransfected TRIM41 with HA-tagged wild type ubiquitin (Ub), Ub with the only lysine residue unchanged (K6O, K11O, K27O, K29O, K33O, K48O, and K63O), or Ub with Lys63 mutated into Arg (K63R). We found that TRIM41 mainly mediated Lys63-linked polyubiquitination of BCL10 (Fig. 5e). In the presence of recombinant Ubc13/Uev1a (putative E2 proteins for Lys63 linkage) and K63O-Ub (but not K63R-Ub), TRIM41 could mediate the polyubiquitination of BCL10 in vitro (Fig. 5f), firmly showing that TRIM41 mediated the Lys63 linkage of BCL10. Then we speculated the Ub-modification sites in BCL10, based on the previous reports.^21,36^ We found that BCL10 with mutations of Lys31 and Lys63 into Arg (BCL10-K31R/K63R) could not be polyubiquitinated by TRIM41 overexpression (Fig. 5g). These data suggest that TRIM41 is an E3 ubiquitin ligase mediating Lys63 linkage of BCL10.

### Lys63 linkage of BCL10 by TRIM41 hubs NEMO for activation of innate antiviral response

BCL10 is indispensable for NF-κB activation in TCR and BCR signaling, possibly via recruitment of NEMO and assembly of the IKK signalosome.^4–6,21,37^ So we speculated that TRIM41-mediated Lys63-linked polyubiquitination of BCL10 may mediate the recruitment of NEMO by providing the Lys63-linked polyubiquitin chains as a handle for recognition by ubiquitin-binding domain (UBA) of NEMO during innate response.

**Fig. 6:**
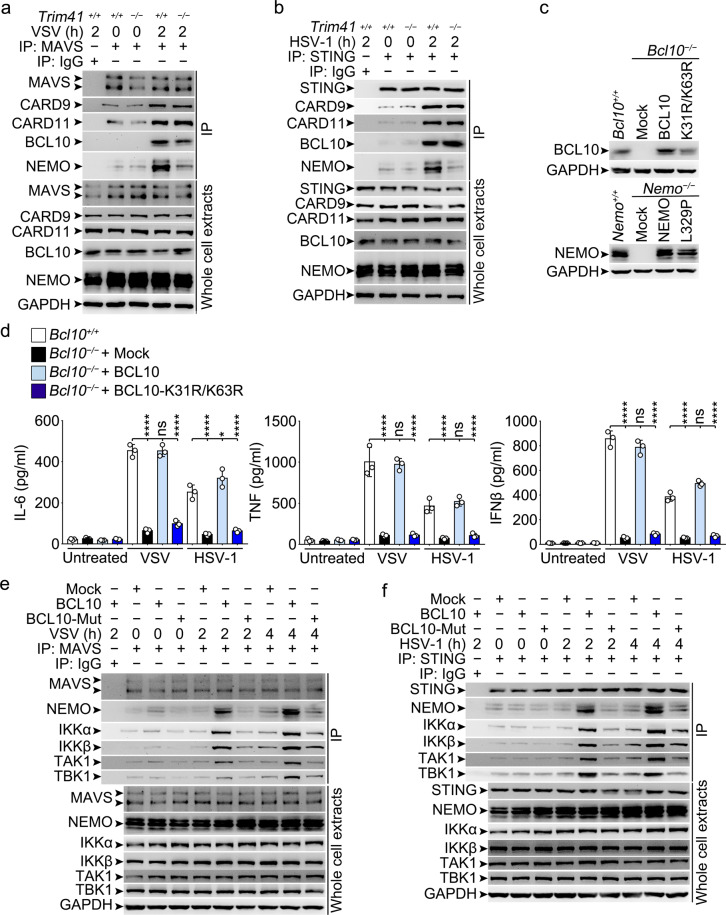
Lys63-linkage of BCL10 by TRIM41 hubs NEMO for activation of virus-triggered innate signaling and response. **a**, **b** *Trim41*^*+/+*^ or *Trim41*^*–/–*^ BMDM cells were infected with VSV (MOI = 1) or HSV-1 (MOI = 5) viruses as indicated. Then whole-cell extracts were immunoprecipitated with anti-MAVS (**a**) or anti-STING (**b**) antibody plus protein A/G beads. Components in the immune complex were examined by Western blotting. **c** Parental (^+/+^) or knockout (^*–/–*^; for *Bcl10* or *Nemo*) RAW264.7 cells were transiently transfected with mock, wild type BCL10 or NEMO, or mutated BCL10 or NEMO vectors as indicated for 48 h. The amounts of BCL10 and NEMO were examined by Western blotting. **d-f** *Bcl10*^*+/+*^ or *Bcl10*^*–/–*^ RAW264.7 cells rescued with mock, WT BCL10 or mutated BCL10 vectors for 48 h as indicated were infected with VSV (MOI = 1) or HSV-1 (MOI = 5) viruses for 8 h (**d**) or as indicated (**e** and **f**). Levels of IL-6, TNF, and IFNβ in supernatants were measured by ELISA (**d**). Otherwise, whole-cell extracts were immunoprecipitated with anti-MAVS (**e**) or anti-STING (**f**) antibody plus protein A/G beads. Components in the immune complex were examined by Western blotting. Results are presented as mean ± SD of triplicates biological replicates (**d**) (One-way ANOVA followed by Bonferroni multiple comparison). One representative experiment of three is shown. ns, not significant; *P < 0.05; ****P < 0.0001

During antiviral innate response, the adaptors as MAVS and STING are serving as platforms for downstream signalosome assembly.^1–3^ We found that TRIM41 could bind with MAVS and STING, which was enhanced by VSV and HSV-1 treatments (Supplementary Fig. S5a, b), indicating that TRIM41 and possibly TRIM41-associated CBM components could be recruited to MAVS and STING. In *Trim41*^*+/+*^ BMDM, CBM components as CARD9, CARD11 and BCL10 as well as NEMO could be detected in MAVS or STING immune complexes (Fig. 6a, b). In *Trim41*^*−/−*^ BMDM, however, we found that the amounts of NEMO coimmunoprecipitated with MAVS and STING after treatments with VSV and HSV-1 respectively were decreased while the recruitment of CBM complex components was not significantly affected (Fig. 6a, b), indicating that the recruitment of NEMO but not CBM components to the MAVS and STING was dependent on TRIM41 and possibly TRIM41-mediated modification of BCL10.

To elucidate the role of Lys63-linked BCL10 in innate response, recruitment of NEMO, and assembly of IKK signalosome, we established *Bcl10*^*−/−*^ RAW264.7 cells and reconstituted the cells with WT BCL10 or BCL10-K31R/K63R (mutant with impaired polyubiquitination; Fig. 6c, upper panel). We found that BCL10 deficiency led to impaired production of IL-6, TNF, and IFNβ in response to VSV and HSV-1 treatments while WT BCL10, but not BCL10-K31R/K63R, could rescue the impaired inflammatory and interferogenic response (Fig. 6d), suggesting that Lys63 linkage of BCL10 was required for innate immune response to cytosolic nucleic acids. In *Bcl10*^*−/−*^ RAW264.7 cells treated with VSV and HSV-1, we found that the amounts of MAVS- and STING-associated NEMO and NEMO-associated kinases (such as IKKα, IKKβ, TAK1, and TBK1) were decreased as compared to that in WT BCL10-reconstituted cells but not in BCL10-K31R/K63R-reconstituted cells (Fig. 6e, f). These data suggest that Lys63 linkage of BCL10 is required for the recruitment of NEMO, assembly of IKK signalosome, and innate immune response to RNA and DNA virus.

### Lys63 linkage of BCL10 and ubiquitin-binding capacity of NEMO are required for TRIM41 in innate antiviral response

**Fig. 7:**
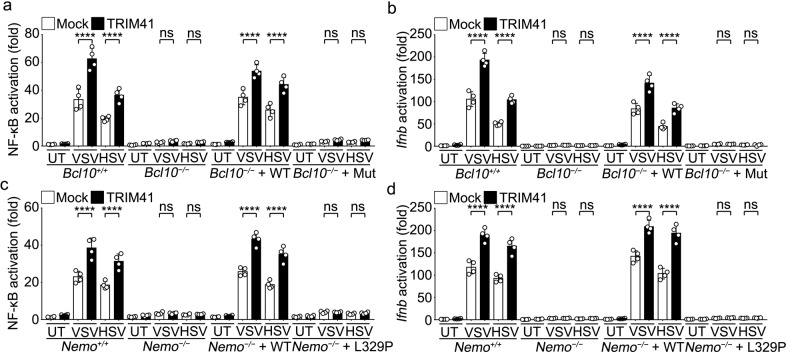
Role of TRIM41 in innate antiviral signaling requires both Lys63-linkage of BCL10 and ubiquitin-binding capacity of NEMO. **a**, **b** *Bcl10*^*+/+*^ or *Bcl10*^*–/–*^ RAW264.7 cells were rescued with mock, WT BCL10, or mutated BCL10 vectors for 48 h as indicated. Then cells were transfected with NF-κB (**a**) or *Ifnb* (**b**) reporters and mock or TRIM41 vectors for 48 h. After infection with VSV (MOI = 1) or HSV-1 (MOI = 5) viruses for 4 h, the luciferase activity was determined. **c**, **d** *Nemo*^*+/+*^ or *Nemo*^*–/–*^ RAW264.7 cells rescued with mock, WT NEMO, or mutated NEMO vectors were transfected with NF-κB (**c**) or *Ifnb* (**d**) reporters and mock or TRIM41 vectors for 48 h. After infection with VSV (MOI = 1) or HSV-1 (MOI = 5) viruses for 4 h, the luciferase activity was determined. Results are presented as mean ± SD of four biological replicates (**a**–**d**) (One-way ANOVA followed by Bonferroni multiple comparison). One representative experiment of three is shown. UT, untreated; HSV, HSV-1; ns, not significant; ****P < 0.0001

Since TRIM41 deficiency caused impaired innate response and decreased Lys63-linked BCL10 polyubiquitination, we hypothesized that the roles of TRIM41 in innate antiviral response may rely on its effects on Lys63-linked polyubiquitylation of BCL10. After overexpression of TRIM41 in *Bcl10*^*+/+*^ and *Bcl10*^*−/−*^ RAW264.7 cells and then treatments with VSV or HSV-1, we found that BCL10 deficiency abrogated TRIM41-mediated enhancement of NF-κB and *Ifnb* gene reporters while the BCL10-K31R/K63R mutant could not rescue the effects of TRIM41 (Fig. 7a, b). Therefore, Lys63-linked polyubiquitylation of BCL10 is crucial for TRIM41-mediated activation of virus-induced innate signaling pathway.

The ubiquitin binding capacity of NEMO has been implicated in control of PRR-triggered both NF-κB and IRF3 activation and thus antiviral innate immune response.^11–13^ Therefore, we established *Nemo*^*−/−*^ RAW264.7 cells and reconstituted the cells with WT NEMO or NEMO with Leu329 substituted with Pro in the UBA domain (NEMO-L329P, incapable of recognizing Ub chains) (Fig. 6c, lower panel). As expected, we found that NEMO deficiency led to impaired production of IL-6, TNF and IFNβ in response to VSV and HSV-1 treatments while WT NEMO, but not NEMO-L329P, could rescue the impaired inflammatory and interferogenic response (Supplementary Fig. S6), suggesting that recognition of polyubiquitin chains by UBA domain of NEMO was required for innate response to viruses. After overexpression of TRIM41 in *Nemo*^*+/+*^ and *Nemo*^*−/−*^ RAW264.7 cells and then treatments with VSV or HSV-1, we found that NEMO deficiency abrogated TRIM41-mediated enhancement of NF-κB and *Ifnb* gene reporters while the NEMO-L329P mutant could not rescue the effects (Fig. 7c, d). Therefore, both the NF-κB activation and the *Ifnb* induction by TRIM41 during innate response to viruses require the ubiquitin-binding capacity of NEMO.

## Discussion

Pathogenic nucleic acids derived from both pathogens and hosts are critical stimulator of innate PRR signaling and have been closely linked to inflammatory, autoimmune and tumorous diseases.^1–3^ Data derived from gene databases, especially data for upregulated *Trim41* mRNA expression in skin samples with lesions derived from psoriasis patients (GEO accession number GSE13355), indicate a potential linkage between *Trim41* and inflammatory diseases. However, genomic variations in *Trim41* has not been reported in inflammatory diseases, but has been deposited in TCGA, Oncomine, and International Cancer Genome Consortium databases for cancer patients. Our study convincingly demonstrates that TRIM41 is critical in innate antiviral response through facilitating pathogenic nucleic acid-triggered signaling pathways after providing genetic and in vivo data, indicating that TRIM41 may be associated with inflammatory and autoimmune human diseases, which, however, need more solid evidence derived from human samples. Whether genetic *Trim41* variations are involved in cancer also need further investigations.

The role and especially the mechanism for TRIM41 in immune response have not been clearly elucidated. It has been reported that TRIM41 may biochemically interact with PKC kinases.^26^ Most recently, TRIM41 is implicated as an E3 ubiquitin ligase for nucleoprotein of influenza A virus and for cGAS in cell models.^27,28^ These studies have not provided solid genetic evidence for TRIM41 in innate response. Our study has illustrated a critical role of TRIM41 in controlling innate immune response to viruses and pathogenic nucleic acids by using *Trim41*^*−/−*^ cells and mice models, thus clearly depicting the immune functions of TRIM41 in innate antiviral response. TRIM41 is a member of the large family of TRIM-containing proteins.^35^ Structurally TRIM41 is similar to TRIM25, which is indispensable for RIG-I activation via Lys63-linked polyubiquitylation of RIG-I following RNA virus infection.^29^ Similar to TRIM25, TRIM41 is also a PRY–SPRY domain-containing TRIM protein and can interact with CARD-containing proteins. However, as compared to the other TRIM proteins, TRIM41 is unique in that TRIM41 is involved in innate response to pathogenic DNA and RNA as well as DNA and RNA viruses, and TRIM41 is the only TRIM protein mediating BCL10 polyubiquitylation. TRIM25 and TRIM31 play crucial roles in innate anti-RNA virus response by polyubiquitinating RIG-I and MAVS respectively while TRIM32 and TRIM56 have been implicated in polyubiquitination of STING.^29–32^ Similar to these E3s, TRIM41 expression levels could be regulated by RNA and DNA viruses, and TRIM41 knockdown could impair innate response to either DNA or RNA viruses, which indicate that TRIM41 may work together with these E3s to tune accurate response to viruses. Possibly due to the common involvement of BCL10 in innate response to both DNA and RNA viruses, TRIM41 can exert a general role in innate antiviral response. In human monocytic U937 cells, TRIM41 was reported to mediate monoubiquitination and activation of cGAS.^27^ In the MS assay of TRIM41-associated proteins, we have detected TRIM41 in protein bands with long-span molecular weight, indicating that TRIM41 itself is usually polyubiquitinated. Whether TRIM41 can regulate PRR-triggered innate response via alternative substrate or different ubiquitin modification type await further studies.

Accumulating studies have revealed the important roles of CBM complex (especially the CARD11-BCL10-MALT1 complex) in adaptive immune response.^4^ However, the roles and mechanisms of CBM complexes in innate immune response have remained largely elusive. Single point mutations of *Card9* and *Card14* have been linked to psoriasis and lung inflammation.^4,38–40^ CARD9, plus BCL10, is essential in anti-fungal, anti-viral, and anti-microbiota innate response.^4,7–9,41^ The CARD9-RAD50 interaction has been implicated in inflammasome activation.^42^ CARD10, together with BCL10, positively regulates inflammatory response to RNA respiratory virus infection.^10^ In keratinocytes, CARD14 plays an important role in inflammatory response.^4,38,39^ In our study, we identified TRIM41 as a CARDs- and BCL10-interacting protein and demonstrated an important role for TRIM41 in polyubiquitinating BCL10, indicating that TRIM41 is a critical and intrinsic component of CBM complex. Whether TRIM41-mediated BCL10 polyubiquitination is universally required for all the CARDs await validation in the future.

Regarding the regulatory machinery for CBM complex in immune response, one important question is how the CBM complex is coupled to immune receptor signaling. In T and B cells, it is proposed that phosphorylation of CARD11 by PKCθ and PKCβ respectively is required for the assembly of CARD11-BCL10-MALT1 complex and the subsequent activation of NF-κB after antigen engagement.^4,43–45^ However, little is known about the coupling of CBM complex to key PRR adaptors. It has been reported CARD10 is inducibly interacting with MAVS after RNA virus infection.^10^ Based on previous reports^4,7–10,46^ and our study, it can be inferred that the CBM complex components, including TRIM41, could be recruited to MAVS and STING adaptors. Whether STING directly serves as adaptor for CBM complex recruitment lacks evidence at present.

An important finding of our study is that TRIM41-mediated Lys63 linkage of BCL10 serves as a hub for NEMO recruitment and is critical in subsequent NEMO-dependent activation of NF-κB and IRF3. In T and B cells, it is reported that UBA domain of NEMO is required for recognition of Lys63-linked BCL10.^21,36^ In THP-1 monocytes and HEK293T cells, NEMO deficient in ubiquitin binding fails to rescue NEMO deficiency-induced impairment of both NF-κB and TBK1-IRF3 activation after RNA virus infection.^12^ So, NEMO may be joined to CBM complex via recognition of ubiquitin chains on CARD proteins, BCL10 or MALT1.^4^ As being implicated in T cells,^21^ BCL10 may be the possible candidate. We found that TRIM41 deficiency did not affect polyubiquitination of CARD proteins but significantly reduced Lys63-linked polyubiquitination of BCL10, which leads to unaffected recruitment of CARDs but almost disappeared recruitment of NEMO to adaptors as MAVS and STING. Therefore we propose that Lys63-linked polyubiquitination of BCL10 is the hub for NEMO during innate antiviral signaling while TRIM41 is the major E3 ubiquitin ligase for Lys63-linked polyubiquitination of BCL10. Our study thus provides mechanistic insights for coupling CBM complex to downstream NEMO and NEMO-associated kinases. Whether TRIM41-mediated BCL10 polyubiquitination also underlies the activation of other immune and inflammatory receptors deserves further investigation.

## Materials and methods

### Mice and cells

6–8 weeks of age C57BL/6 (H-2K^b^) WT mice were purchased from Joint Ventures Sipper BK Experimental Animal (Shanghai, China). The *Trim41* complete knockout mice were established with the assistance of the Shanghai Model Organisms Center & Inc. (Shanghai, China) as described,^24^ and were backcrossed with C57BL/6 mice for more than six generations for experiments. For genotyping of mice, tail DNA was extracted, and PCR was performed as described.^24^ The genotyping primer sequences and the standards were described in Supplementary Table S2. All animal experiments were undertaken in accordance with the National Institute of Health Guide for the Care and Use of Laboratory Animals, with the approval of the Scientific Investigation Board of Second Military Medical University, Shanghai. HEK293T and RAW264.7 cells were obtained from and authenticated by the American Type Culture Collection (Manassas, VA), and were cultured as suggested by the supplier. PM, BMDC, BMDM, CD4^+^ or CD8^+^ splenic T cells, and CD19^+^ splenic B cells were isolated or prepared and cultured as described previously.^23–25^ For the depletion of mouse BCL10 and NEMO in RAW264.7 cells, pc3-U6-guide RNA-CMV-RED (encoding guiding RNA and red fluorescent protein) and Cas9-IRES-EGFP (encoding Cas9 and green fluorescent protein) plasmids (kind gifts from Shanghai Model Organisms Center & Inc, Shanghai, China) were cotransfected into RAW264.7 cells as described.^25^ Cells with both red and green fluorescence were then sorted by using Gallios Flow Cytometer (Beckman Coulter, Brea, CA). Sorted cells were cultured for 3–5 days, and clones propagated from single-cell were picked out. Four target sequences for guiding RNA synthesis against *Bcl10* or *Nemo* were designed (Supplementary Table S2).

### Antibodies and reagents

The antibodies used in this study were listed in Supplementary Table S3. Recombinant Ubiquitin and derivatives were from Boston Biochem Inc. (Cambridge, MA). Recombinant CARD9 (ab131800) and BCL10 (ab82241) were obtained from Abcam Inc. (Cambridge, MA). Recombinant CARD11 was obtained from Creative BioMart (Shirley, NY; Card11-385M). Other non-specified reagents were purchased from Sigma-Aldrich (St. Louis, MO).

### Plasmids, transfection, and RNA interference

The recombinant vectors encoding mouse TRIM41 (GenBank No. NM_145377.2), BCL10 (GenBank No. NM_009740.2), NEMO (GenBank No. NM_001161423.1), and the indicated mutations were constructed as described.^23–25^ For transient transfection of plasmids in RAW264.7 and HEK293T cells, the X-tremeGENE HP reagents were used according to manufacturer’s instructions (Roche, Welwyn Garden City, UK). For transient knockdown of *Trim41*, three siRNA duplexes were synthesized (Supplementary Table S2) and transfected using the INTERFERin-HTS according to the standard protocol (Polyplus-transfection Company, Illkirch, France). The siRNA duplexes specific for *Adar1* (sc-37658) and *Trex1* (sc-63158) were obtained from Santa Cruz Biotechnology (Dallas, TX). The non-sense sequence 5′-TTCTCCGAACGTGTCACG-3′ was used as control siRNA.

### Quantitative-PCR

Total cellular RNA was extracted using Trizol reagent (Invitrogen Corporation, CA, USA), and cDNA was synthesized by using the AMV Reverse Transcriptase kit (Promega, Madison, WI). The mRNA quantification methods were as described.^23,24^ The primer sequences were listed in Supplementary Table S2

### ELISA assay of cytokines

ELISA kits for mouse IFNβ, TNF, and IL-6 were from R&D Systems (Minneapolis, MN). The concentrations of cytokines in the culture supernatants or serum were determined as recommended by the manufacturer.

### Nanospray liquid chromatography–tandem mass spectrometry

The identification of the TRIM41-associated proteins was performed as described previously.^24,25^ The nano-ultra performance liquid chromatography–electrospray ionization tandem MS was performed by the Beijing Genomics Institute (Beijing, China).

### Luciferase assays

The NF-κB and *Ifnb* luciferase reporter plasmids were obtained from Panomics of Affymetrix (Santa Clara, CA) or as described.^25^ Luciferase activities were measured with Dual-Luciferase Reporter Assay System (Promega, Madison, WI). The determination of reporter transactivation was performed as described previously.^25^

### Immunoprecipitation and immunoblot

The immunoprecipitations using anti-TRIM41, anti-MAVS, anti-STING, anti-Flag, or anti-Myc antibodies and the immunoblot assays were performed as described previously.^23–25^ The polyubiquitination assay were performed as described previously.^22–25^

### GST pull-down assays

The GST pull-down assays using GST or GST-TRIM41 were as described previously.^22–25^

### Immunofluorescence confocal microscopy

RAW264.7 cells seeded on cover sliders were transiently co-transfected with TRIM41-GFP and BCL10-RFP vectors for 48 h. Samples were washed briefly in PBS and fixed in 4% paraformaldehyde. Upon confocal microscopy, cells were immediately stained with 10 μg/ml DAPI and covered by cover glasses. Images were obtained with a laser scanning confocal microscope (Leica TCS SP8) and analyzed by the LAS X software version 2.0.2.15022.

### Animal models and manipulations

Age- and gender-matched mice with indicated genotype were used to establish animal models. For in vivo infection with pathogens, mice were intraperitoneally injected with 100 μl PBS containing 5 × 10^6^ PFUs of VSV or LM, or 2 × 10^8^ PFUs of HSV-1. The microbe titers in the liver or spleen were determined by plaque formation assays as described previously.^22^ To mimic the neural infection of HSV-1, 100 μl PBS containing 2 × 10^8^ PFUs of HSV-1 was intravenously injected via tail vein. The replication of HSV-1 in the brain was evaluated by immunohistochemistry assay of HSV1 protein or by Q-PCR assay of *Icp0* mRNA.

### Statistical analysis

All the experiments were independently repeated at least two or three times. Results are given as mean ± SE or mean ± SD. Comparisons between two groups were done using unpaired Student’s t-test. The survival curve was established by the Kaplan and Meier method, and analyzed by Log-rank test. Multiple comparisons were done with one-way ANOVA followed by Bonferroni multiple comparisons. Statistical significance was determined as *P* < 0.05.

## Supplementary information

TRIM41 is required to innate antiviral response by polyubiquitinating BCL10 and recruiting NEMO

Dataset 1

Dataset 2

## Data Availability

The authors declare that there are no primary datasets and computer codes associated with this study. All data and materials are available to the researchers once published.

## References

[1] Cao X (2016). Self-regulation and cross-regulation of pattern-recognition receptor signalling in health and disease. Nat. Rev. Immunol..

[2] Yin Q, Fu TM, Li J, Wu H (2015). Structural biology of innate immunity. Annu. Rev. Immunol..

[3] Crowl JT, Gray EE, Pestal K, Volkman HE, Stetson DB (2017). Intracellular nucleic acid detection in autoimmunity. Annu. Rev. Immunol..

[4] Ruland J, Hartjes L (2019). CARD-BCL-10-MALT1 signalling in protective and pathological immunity. Nat. Rev. Immunol..

[5] Ruland J (2001). Bcl10 is a positive regulator of antigen receptor-induced activation of NF-kappaB and neural tube closure. Cell.

[6] Xue L (2003). Defective development and function of Bcl10-deficient follicular, marginal zone and B1 B cells. Nat. Immunol..

[7] Gross O (2006). Card9 controls a non-TLR signalling pathway for innate anti-fungal immunity. Nature.

[8] Hara H (2007). The adaptor protein CARD9 is essential for the activation of myeloid cells through ITAM-associated and Toll-like receptors. Nat. Immunol..

[9] Hsu YM (2007). The adaptor protein CARD9 is required for innate immune responses to intracellular pathogens. Nat. Immunol..

[10] Jiang C (2016). CARMA3 is a host factor regulating the balance of inflammatory and antiviral responses against viral infection. Cell Rep..

[11] Fang R (2017). NEMO-IKKβ are essential for IRF3 and NF-κB activation in the cGAS-STING pathway. J. Immunol..

[12] Fang R (2017). MAVS activates TBK1 and IKKε through TRAFs in NEMO dependent and independent manner. PLoS Pathog..

[13] Zhao T (2007). The NEMO adaptor bridges the nuclear factor-kappaB and interferon regulatory factor signaling pathways. Nat. Immunol..

[14] Zhou H (2004). Bcl10 activates the NF-kappaB pathway through ubiquitination of NEMO. Nature.

[15] Lobry C, Lopez T, Israël A, Weil R (2007). Negative feedback loop in T cell activation through IkappaB kinase-induced phosphorylation and degradation of Bcl10. Proc. Natl Acad. Sci. USA.

[16] Shinohara H, Maeda S, Watarai H, Kurosaki T (2007). IkappaB kinase beta-induced phosphorylation of CARMA1 contributes to CARMA1-Bcl10-MALT1 complex formation in B cells. J. Exp. Med..

[17] Wegener E (2006). Essential role for IkappaB kinase beta in remodeling Carma1-Bcl10-Malt1 complexes upon T cell activation. Mol. Cell.

[18] Zeng H (2007). Phosphorylation of Bcl10 negatively regulates T-cell receptor-mediated NF-kappaB activation. Mol. Cell. Biol..

[19] Hu S (2006). cIAP2 is a ubiquitin protein ligase for BCL10 and is dysregulated in mucosa-associated lymphoid tissue lymphomas. J. Clin. Invest..

[20] Scharschmidt E, Wegener E, Heissmeyer V, Rao A, Krappmann D (2004). Degradation of Bcl10 induced by T-cell activation negatively regulates NF-kappa B signaling. Mol. Cell. Biol..

[21] Wu CJ, Ashwell JD (2008). NEMO recognition of ubiquitinated Bcl10 is required for T cell receptor-mediated NF-kappaB activation. Proc. Natl Acad. Sci. USA.

[22] Wang C (2009). The E3 ubiquitin ligase Nrdp1 ‘preferentially’ promotes TLR-mediated production of type I interferon. Nat. Immunol..

[23] Yang M (2011). E3 ubiquitin ligase CHIP facilitates Toll-like receptor signaling by recruiting and polyubiquitinating Src and atypical PKC{zeta}. J. Exp. Med..

[24] Yang M (2015). K33-linked polyubiquitination of Zap70 by Nrdp1 controls CD8(+) T cell activation. Nat. Immunol..

[25] Yu Z (2016). Lys29-linkage of ASK1 by Skp1-Cullin 1-Fbxo21 ubiquitin ligase complex is required for antiviral innate response. Elife.

[26] Chen D (2007). Amplitude control of protein kinase C by RINCK, a novel E3 ubiquitin ligase. J. Biol. Chem..

[27] Liu ZS (2018). RINCK-mediated monoubiquitination of cGAS promotes antiviral innate immune responses. Cell Biosci..

[28] Patil G (2018). TRIM41-mediated ubiquitination of nucleoprotein limits Influenza A virus infection. J. Virol..

[29] Gack MU (2007). TRIM25 RING-finger E3 ubiquitin ligase is essential for RIG-I-mediated antiviral activity. Nature.

[30] Liu B (2017). The ubiquitin E3 ligase TRIM31 promotes aggregation and activation of the signaling adaptor MAVS through Lys63-linked polyubiquitination. Nat. Immunol..

[31] Zhang J, Hu MM, Wang YY, Shu HB (2012). TRIM32 protein modulates type I interferon induction and cellular antiviral response by targeting MITA/STING protein for K63-linked ubiquitination. J. Biol. Chem..

[32] Tsuchida T (2010). The ubiquitin ligase TRIM56 regulates innate immune responses to intracellular double-stranded DNA. Immunity.

[33] Hartner JC, Walkley CR, Lu J, Orkin SH (2009). ADAR1 is essential for the maintenance of hematopoiesis and suppression of interferon signaling. Nat. Immunol..

[34] Yang YG, Lindahl T, Barnes DE (2007). Trex1 exonuclease degrades ssDNA to prevent chronic checkpoint activation and autoimmune disease. Cell.

[35] van Gent M, Sparrer KMJ, Gack MU (2018). TRIM proteins and their roles in antiviral host defenses. Annu. Rev. Virol..

[36] Yang Y (2016). Targeting non-proteolytic protein ubiquitination for the treatment of diffuse large B cell lymphoma. Cancer Cell.

[37] Sun L, Deng L, Ea CK, Xia ZP, Chen ZJ (2004). The TRAF6 ubiquitin ligase and TAK1 kinase mediate IKK activation by BCL10 and MALT1 in T lymphocytes. Mol. Cell.

[38] Jordan CT (2012). PSORS2 is due to mutations in CARD14. Am. J. Hum. Genet..

[39] Jordan CT (2012). Rare and common variants in CARD14, encoding an epidermal regulator of NF-kappaB, in psoriasis. Am. J. Hum. Genet..

[40] Xu X (2018). CARD9S12N facilitates the production of IL-5 by alveolar macrophages for the induction of type 2 immune responses. Nat. Immunol..

[41] Lamas B (2016). CARD9 impacts colitis by altering gut microbiota metabolism of tryptophan into aryl hydrocarbon receptor ligands. Nat. Med..

[42] Roth S (2014). Rad50-CARD9 interactions link cytosolic DNA sensing to IL-1β production. Nat. Immunol..

[43] Sommer K (2005). Phosphorylation of the CARMA1 linker controls NF-kappaB activation. Immunity.

[44] Matsumoto R (2005). Phosphorylation of CARMA1 plays a critical role in T Cell receptor-mediated NF-kappaB activation. Immunity.

[45] Shinohara H (2005). PKC beta regulates BCR-mediated IKK activation by facilitating the interaction between TAK1 and CARMA1. J. Exp. Med..

[46] Wang X (2016). STING requires the adaptor TRIF to trigger innate immune responses to microbial infection. Cell Host Microbe.

