# Supplementary material for: TRIM41 is required to innate antiviral response by polyubiquitinating BCL10 and recruiting NEMO
Source: Signal Transduct Target Ther. 2021 Feb 28;6:90. doi: 10.1038/s41392-021-00477-8 (PMC7914255; doi:10.1038/s41392-021-00477-8)
Supplement: Supplementary file 1 — TRIM41 is required to innate antiviral response by polyubiquitinating BCL10 and recruiting NEMO [file 41392_2021_477_MOESM1_ESM.pdf]

Supplementary data for

TRIM41 is required to innate antiviral response by polyubiquitinating BCL10 and recruiting NEMO

Zhou Yu[1,2,3,†], Xuelian Li[3,4,†], Mingjin Yang[3,†], Jiaying Huang[4], Qian Fang[3], Jianjun Jia[3], Zheng Li[3], Yan Gu[3], Taoyong Chen[3,*] & Xuetao Cao[1,2,3,4,5,*]

[1] Center for Systems Medicine, Department of Immunology, Institute of Basic Medical Sciences, Peking Union Medical College, Chinese Academy of Medical Sciences, Beijing 100005, China

[2] Suzhou Institute of Systems Medicine, Suzhou 215123, Jiangsu, China

[3] National Key Laboratory of Medical Immunology & Institute of Immunology, Second Military Medical University, Shanghai 200433, China

[4] Institute of Immunology, Zhejiang University School of Medicine, Hangzhou 310058, China

[5] College of Life Science, Nankai University, Tianjin 300071, China

[†] These authors contributed equally to this work.

* Corresponding author. E-mail: chenty@immunol.org (T.C.); caoxt@immunol.org (X.C.)

This PDF include:

- Table S1-S3
- Figures S1-S6

Other Supplementary Materials for this manuscript include the following:

- Source data for Figures (PDF document)
- Source data for Graphs (Excel document)

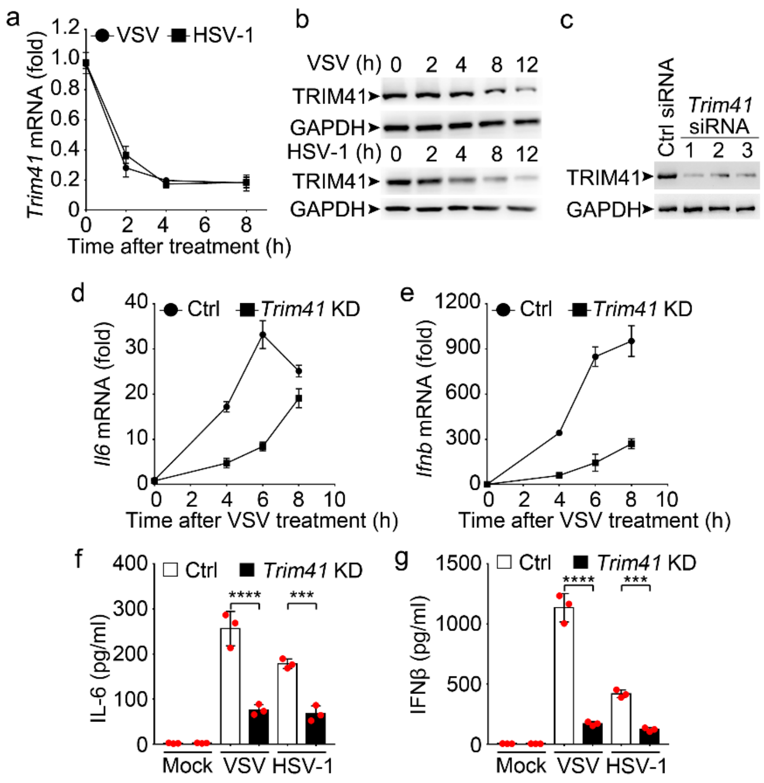

Figure S1. Knockdown of *Trim41* impairs innate immune response against VSV in peritoneal macrophages. a, **b** Peritoneal macrophages from C57BL/6 mice were infected with VSV (MOI = 1) or HSV-1 (MOI = 5). The mRNA levels of *Trim41* were evaluated by Q-PCR (**a**). The protein levels of TRIM41 were evaluated by Western blotting (**b**). **c-g** Peritoneal macrophages from C57BL/6 mice were transfected with control (Ctrl) or *Trim41*-specific siRNAs for 48h. The efficiency of knockdown (KD) was evaluated by Western blotting (**c**). The cells were then infected with VSV (MOI = 1) or HSV-1 (MOI = 5) as indicated (**d** and **e**) or for 8h (**f** and **g**). The mRNA levels of *Il6* and *Ifnb* were evaluated by Q-PCR (**d** and **e**). Amounts of IL-6 and IFNβ in the supernatants were measured by ELISA (**f** and **g**). Results are presented as mean ± SD of triplicate samples (**a** and **d-g**; one-way ANOVA followed by Bonferroni multiple comparison). One representative data of three independent experiments are shown. ***, $P < 0.001$; ****, $P < 0.0001$.

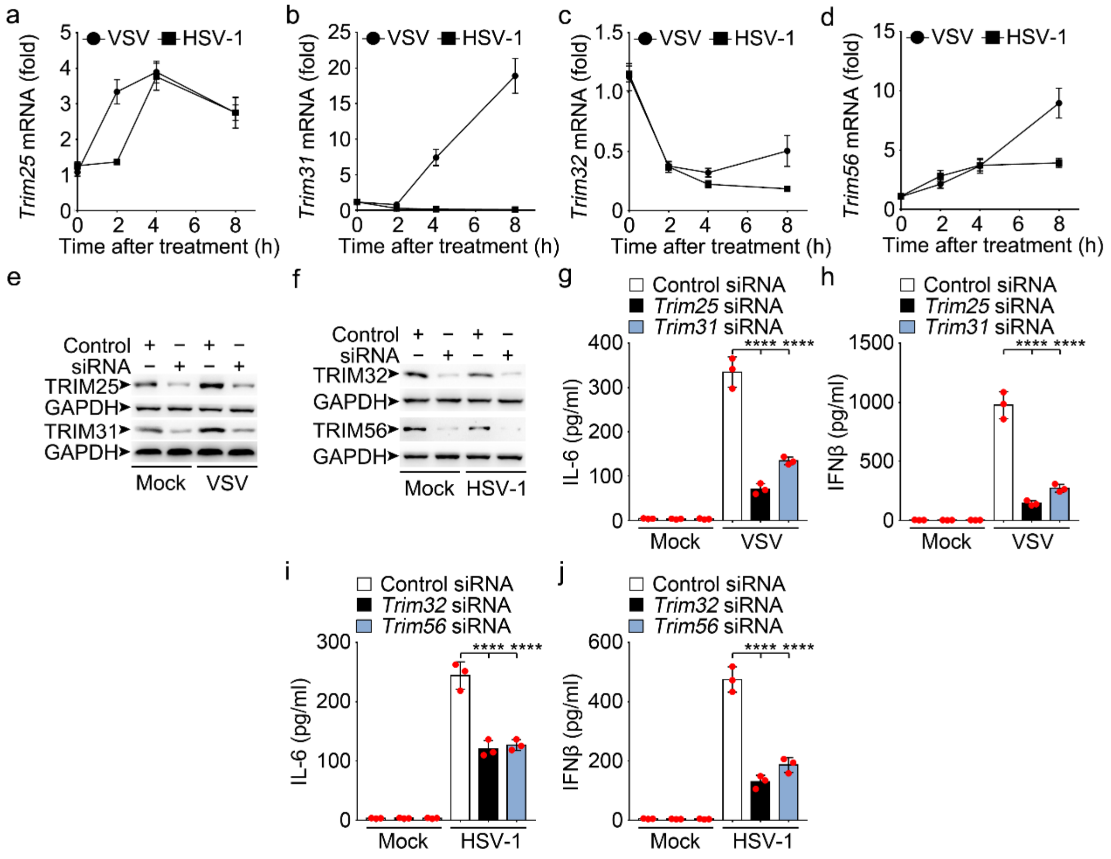

Figure S2. Confirmation for the role of key TRIM proteins in innate antiviral response, for comparison with TRIM41. a-d Peritoneal macrophages from C57BL/6 mice were infected with VSV (MOI = 1) or HSV-1 (MOI = 5). The mRNA levels of *Trim25* (**a**), *Trim31* (**b**), *Trim32* (**c**) or *Trim56* (**d**) were evaluated by Q-PCR. **e-j** Peritoneal macrophages from C57BL/6 mice were transfected with control (Ctrl) or *Trim*-specific siRNAs as indicated for 48h and then infected with VSV (MOI = 1) or HSV-1 (MOI = 5) for 8h. The efficiency of knockdown was evaluated by Western blotting (**e** and **f**). Amounts of IL-6 and IFNβ in the supernatants were measured by ELISA (**g-j**). Results are presented as mean ± SD of triplicate samples (**a-d** and **g-j**; one-way ANOVA followed by Bonferroni multiple comparison). One representative data of three independent experiments are shown. ****, $P < 0.0001$.

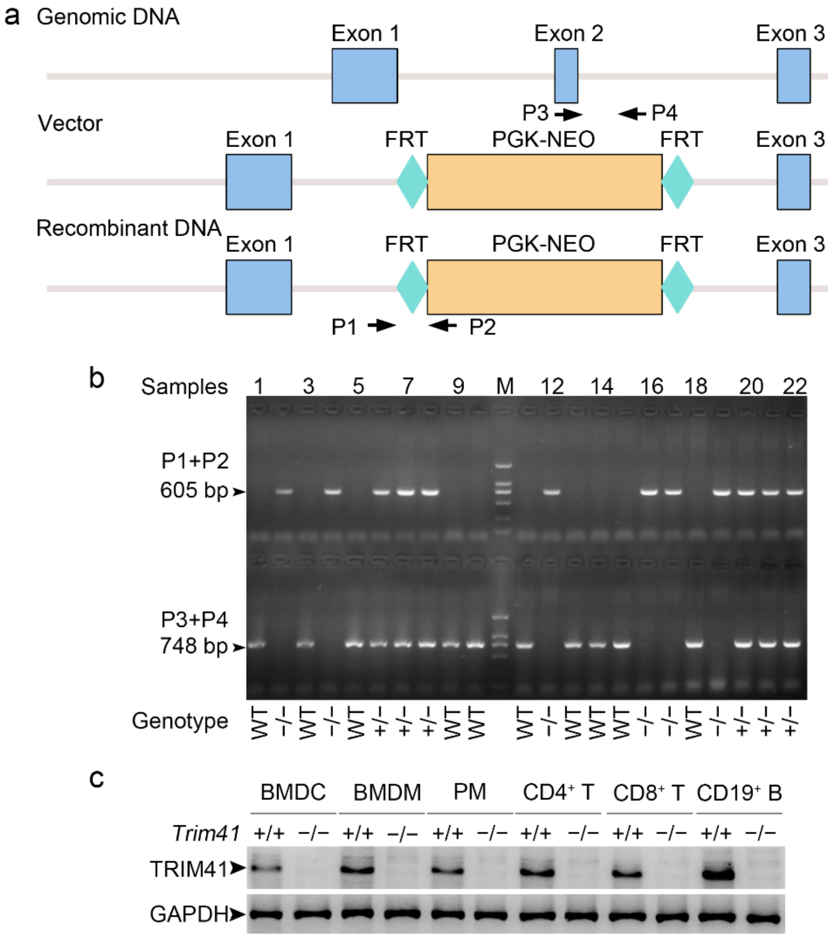

Figure S3. Establishment of *Trim41* knockout mice. a Strategy to establish *Trim41*$^{-/-}$ mice. **b** PCR assays of mice tail DNA for identification of *Trim41*$^{-/-}$ mice. M, DNA marker. **c** Western blotting assays of TRIM41 amounts in bone marrow-derived dendritic cells (BMDC), bone marrow-derived macrophages (BMDM), peritoneal macrophages (PM), or splenic CD4^{+} T cells, CD8^{+} T cells or CD19^{+} B cells.

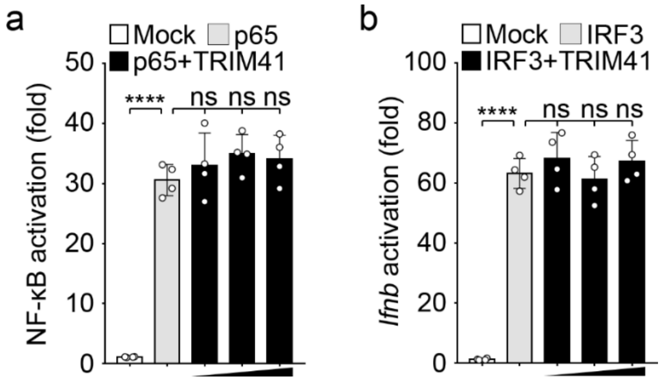

Figure S4. TRIM41 does not affect p65-mediated or IRF3-mediated activation of NF-κB and *Ifnb* reporters. a, **b** HEK293T cells were cotransfected with mock, Flag-tagged TRIM41 (10, 50 or 100 ng), p65 (100 ng) or IRF3 (100 ng) vectors and indicated reporter vectors. 48h later, the luciferase activity was determined. Results are presented as mean ± SD of four biological samples (one-way ANOVA followed by Bonferroni multiple comparison). One representative experiment of three is shown. ns, not significant; ****, $P < 0.0001$.

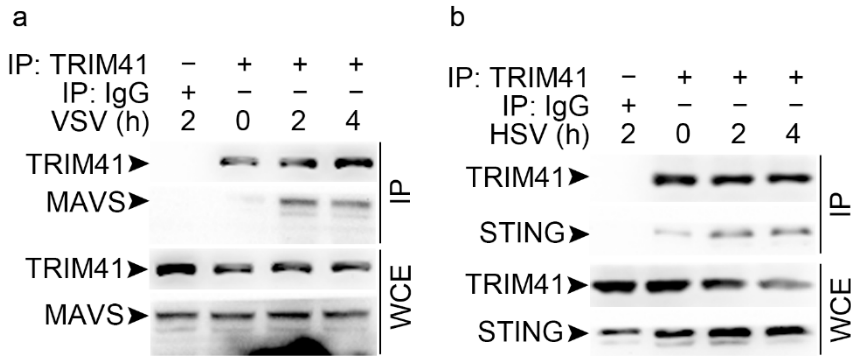

Figure S5. Endogenous TRIM41 interacts with MAVS and STING. a, **b** Wild type BMDM cells were infected with VSV (MOI = 1) or HSV-1 (MOI = 5) viruses as indicated. Then whole cell extracts (WCE) were immunoprecipitated with anti-TRIM41 antibody plus protein A/G beads. Components in the TRIM41 complex were examined by Western blotting. One representative experiment of three is shown.

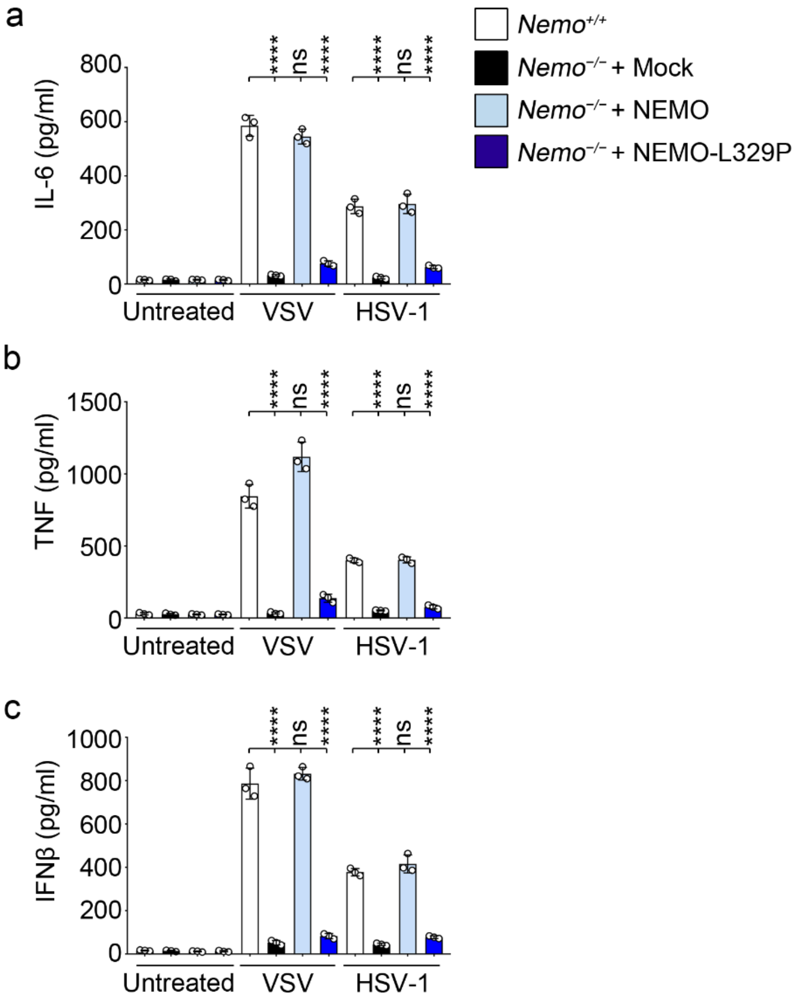

Figure S6. Ubiquitin-binding domain of NEMO is required for innate antiviral response. a-c *Nemo*$^{+/+}$ or *Nemo*$^{-/-}$ RAW264.7 cells rescued with mock, wild type NEMO or mutated NEMO vectors for 48h as indicated were infected with VSV (MOI = 1) or HSV-1 (MOI = 5) viruses for 8h. Levels of IL-6 (**a**), TNF (**b**) and IFNβ (**c**) in supernatants were measured by ELISA. Results are presented as mean ± SD of biological triplicates (One-way ANOVA followed by Bonferroni multiple comparison). One representative experiment of three is shown. ns, not significant; ****, $p < 0.0001$.

Table S1. TRIM41-associated proteins identified by mass spectrometry. [a,b]

Sample range	Protein Accession No.	Protein name	Protein definition	Peptide count	Unique peptide count	Cover percent	Molecular weight
SR1 (>170 kDa)	Q8WV44	TRIM41	E3 ubiquitin-protein ligase TRIM41[c]	6	4	6.03%	71670.38
	Q9NTJ3	SMC4	Structural maintenance of chromosomes protein 4	2	2	1.79%	147182.07
	P07814	EPRS	Bifunctional glutamate/proline--tRNA ligase	2	2	1.46%	170590.85
	P62987	UBA52	Ubiquitin-60S ribosomal protein L40	1	1	10.16%	14728.26
	P62979	RPS27A	Ubiquitin-40S ribosomal protein S27a	1	1	8.33%	17964.86
	P0CG47	UBB	Polyubiquitin-B	1	1	5.68%	25761.57
	P0CG48	UBC	Polyubiquitin-C	1	1	1.90%	77038.42
	Q9BRJ2	MRPL45	39S ribosomal protein L45, mitochondrial	1	1	3.27%	35350.95
	Q00325	SLC25A3	Phosphate carrier protein, mitochondrial	1	1	3.31%	40094.92
	P62805	HIST1H4 A	Histone H4	1	1	9.71%	11367.28
	Q14152	EIF3A	Eukaryotic translation initiation factor 3 subunit A	1	1	0.58%	166570.47
SR2 (>130 kDa)	P42704	LRPPRC	Leucine-rich PPR motif-containing protein, mitochondrial	2	2	1.51%	157905.35
	P05109	S100A8	Protein S100-A8	1	1	11.83%	10834.49
	O95347	SMC2	Structural maintenance of chromosomes protein 2	1	1	0.92%	135655.9
	O14974	PPP1R12 A	Protein phosphatase 1 regulatory subunit 12A	1	1	0.97%	115280.86
	Q4VCS5	AMOT	Angiomotin	1	1	1.38%	118085.85
	O95785	WIZ	Protein Wiz	1	1	0.73%	178675.23
	P05023	ATP1A1	Sodium/potassium-transporting ATPase subunit alpha-1	1	1	1.27%	112896.13
SR3 (～110-130 kDa)	P52732	KIF11	Kinesin-like protein KIF11	18	9	10.42%	119158.9
	Q13263	TRIM28	Transcription intermediary factor 1-beta	7	4	6.47%	88549.91

Q8WV44	TRIM41	E3 ubiquitin-protein ligase TRIM41	4	3	5.40%	71670.38
Q9HCE1	MOV10	Putative helicase MOV-10	2	2	2.79%	113671.61
P68104	EEF1A1	Elongation factor 1-alpha 1	2	1	2.38%	50140.69
Q05639	EEF1A2	Elongation factor 1-alpha 2	2	1	2.38%	50470.03
Q5VTE0	EEF1A1P5	Putative elongation factor 1-alpha-like 3	2	1	2.38%	50184.85
P06576	ATP5B5	ATP synthase subunit beta, mitochondrial	2	1	2.08%	56559.96
Q96AV8	E2F7	Transcription factor E2F7	2	1	1.32%	99888.43
Q9H0D6	XRN2	5'-3' exoribonuclease 2	2	1	1.26%	108582.88
Q92685	ALG3	Dol-P-Man:Man(5)GlcNAc(2)-PP-Dol alpha-1,3-mannosyltransferase	2	1	1.83%	50126.55
P05023	ATP1A1	Sodium/potassium-transporting ATPase subunit alpha-1	2	1	1.27%	112896.13
P62736	ACTA2	Actin, aortic smooth muscle	1	1	2.65%	42009.01
P60709	ACTB	Actin, cytoplasmic 1	1	1	2.67%	41736.77
P68032	ACTC1	Actin, alpha cardiac muscle 1	1	1	2.65%	42019.01
P63261	ACTG1	Actin, cytoplasmic 2	1	1	2.67%	41792.89
P63267	ACTG2	Actin, gamma-enteric smooth muscle	1	1	2.66%	41876.93
P68133	ACTA1	Actin, alpha skeletal muscle	1	1	2.65%	42051.06
P55884	EIF3B	Eukaryotic translation initiation factor 3 subunit B	1	1	1.60%	92481.88
P62987	UBA52	Ubiquitin-60S ribosomal protein L40	1	1	10.16%	14728.26
P62979	RPS27A	Ubiquitin-40S ribosomal protein S27a	1	1	8.33%	17964.86
P0CG47	UBB	Polyubiquitin-B	1	1	5.68%	25761.57
P0CG48	UBC	Polyubiquitin-C	1	1	1.90%	77038.42
J3QLP7	UBBP4	Protein UBBP4	1	1	5.80%	25153.98
Q2TAL8	QRICH1	Glutamine-rich protein 1	1	1	1.42%	86435.67
O00410	IPO5	Importin-5	1	1	1.00%	123630.13

	O15084	ANKRD28	Serine/threonine-protein phosphatase 6 regulatory ankyrin repeat subunit A	1	1	1.42%	112965.69
	P25705	ATP5A1	ATP synthase subunit alpha, mitochondrial	1	1	2.35%	59750.67
	P33176	KIF5B	Kinesin-1 heavy chain	1	1	1.56%	109684.8
	O00748	CES2	Cocaine esterase	1	1	1.97%	61806.95
	Q9BXL6	CARD14	Caspase recruitment domain-containing protein 14	1	1	1.20%	113270.18
	Q6UB99	ANKRD11	Ankyrin repeat domain-containing protein 11	1	1	0.26%	297914.52
	Q71RC2	LARP4	La-related protein 4	1	1	1.38%	80596.16
	Q9UDR5	AASS	Alpha-aminoadipic semialdehyde synthase, mitochondrial	1	1	1.08%	102131.84
	Q9HD23	MRS2	Magnesium transporter MRS2 homolog, mitochondrial	1	1	2.03%	50317.81
	Q14444	CAPRIN1	Caprin-1	1	1	1.55%	78366.36
	Q86YZ3	HRNR	Hornerin	1	1	0.35%	282390.98
	P09874	PARP1	Poly [ADP-ribose] polymerase 1	1	1	1.28%	113083.66
SR4 (～90-100 kDa)	Q8WV44	TRIM41	E3 ubiquitin-protein ligase TRIM41	142	18	17.94%	71670.38
	P52732	KIF11	Kinesin-like protein KIF11	8	5	5.11%	119158.9
	P23246	SFPQ	Splicing factor, proline- and glutamine-rich	6	2	3.54%	76149.73
	P13639	EEF2	Elongation factor 2	3	2	2.91%	95338.18
	Q5VUJ6	LRCH2	Leucine-rich repeat and calponin homology domain-containing protein 2	2	2	1.70%	84588.5
	Q6P3W7	SCYL2	SCY1-like protein 2	2	2	3.01%	103708.49
	Q13200	PSMD2	26S proteasome non-ATPase regulatory subunit 2	3	1	1.65%	100199.83
	Q7Z2W4	ZC3HAV1	Zinc finger CCCH-type antiviral protein 1	2	1	1.66%	101431.46
	P56192	MARS	Methionine--tRNA ligase, cytoplasmic	2	1	1.11%	101115.94
	Q9Y5V3	MAGED1	Melanoma-associated antigen D1	2	1	1.41%	86161

	A8MWS5	NBP6L	Putative neuroblastoma breakpoint family member 6-like protein	1	1	6.67%	27623.25
	Q8NF91	SYNE1	Nesprin-1	1	1	0.18%	1011087.5
	O75153	CLUH	Clustered mitochondria protein homolog	1	1	1.15%	146670.27
	Q8N0Z6	TTC5	Tetratricopeptide repeat protein 5	1	1	3.41%	48927.57
	Q9NR30	DDX21	Nucleolar RNA helicase 2	1	1	1.15%	87344.38
	Q658Y4	FAM91A1	Protein FAM91A1	1	1	1.67%	93909.12
	Q96FX9	CLDN15	Claudin 15	1	1	9.38%	13777.99
	Q5C9Z4	NOM1	Nucleolar MIF4G domain-containing protein 1	1	1	1.28%	96257.65
	Q9Y4D8	HECTD4	Probable E3 ubiquitin-protein ligase HECTD4	1	1	0.30%	439344.9
	P05023	ATP1A1	Sodium/potassium-transporting ATPase subunit alpha-1	1	1	1.27%	112896.13
	P12814	ACTN1	Alpha-actinin-1	1	1	1.35%	103057.69
SR5 (~90 kDa)	Q8WV44	TRIM41	E3 ubiquitin-protein ligase TRIM41	47	9	11.90%	71670.38
	Q9BRS2	RIOK1	Serine/threonine-protein kinase RIO1	25	9	17.08%	65582.95
	P08238	HSP90AB1	Heat shock protein HSP 90-beta	12	5	8.84%	83264.38
	P52732	KIF11	Kinesin-like protein KIF11	6	4	4.45%	119158.9
	Q8N1F7	NUP93	Nuclear pore complex protein Nup93	3	3	4.15%	93488.56
	P33992	MCM5	DNA replication licensing factor MCM5	3	2	3.27%	82285.73
	O75534	CSDE1	Cold shock domain-containing protein E1	3	2	2.76%	88884.63
	Q01844	EWSR1	RNA-binding protein EWS	2	1	2.13%	68478.32
	Q16891	IMMT	Mitochondrial inner membrane protein	2	1	1.72%	83678.12
	P50416	CPT1A	Carnitine O-palmitoyltransferase 1, liver isoform	2	1	1.81%	88367.71
	Q9P291	ARMCX1	Armadillo repeat-containing X-linked protein 1	1	1	2.65%	49180.34
	P62987	UBA52	Ubiquitin-60S ribosomal protein L40	1	1	10.16%	14728.26
	P62979	RPS27A	Ubiquitin-40S ribosomal protein S27a	1	1	8.33%	17964.86

	P0CG47	UBB	Polyubiquitin-B	1	1	5.68%	25761.57
	P0CG48	UBC	Polyubiquitin-C	1	1	1.90%	77038.42
	J3QLP7	UBBP4	Protein UBBP4	1	1	5.80%	25153.98
	Q9P2Z0	THAP10	THAP domain-containing protein 10	1	1	3.89%	28350.85
	Q12906	ILF3	Interleukin enhancer-binding factor 3	1	1	1.57%	95338.39
	Q8IXB1	DNAJC10	homolog subfamily C member 10	1	1	1.39%	91079.75
	O14641	DVL2	Segment polarity protein dishevelled homolog DVL-2	1	1	1.63%	78948.31
	Q7Z736	PLEKHH3	Pleckstrin homology domain-containing family H member 3	1	1	1.51%	85317.88
	Q92499	DDX1	ATP-dependent RNA helicase DDX1	1	1	2.16%	82432.04
	Q8N485	LIX1	Protein limb expression 1 homolog	1	1	2.48%	31891.48
	P49959	MRE11A	Double-strand break repair protein MRE11A	1	1	1.69%	80593.4
	Q14562	DHX8	ATP-dependent RNA helicase DHX8	1	1	0.82%	139314.72
	Q92620	DHX38	Pre-mRNA-splicing factor ATP-dependent RNA helicase PRP16	1	1	0.81%	140503.2
	Q86YZ3	HRNR	Hornerin	1	1	0.35%	282390.98
	Q9P2D8	UNC79	Protein unc-79 homolog	1	1	0.61%	295313.15
SR6 (～80 kDa)	Q8WV44	TRIM41	E3 ubiquitin-protein ligase TRIM41	18	7	9.84%	71670.38
	Q9BRS2	RIOK1	Serine/threonine-protein kinase RIO1	17	7	14.44%	65582.95
	P23588	EIF4B	Eukaryotic translation initiation factor 4B	12	7	12.27%	69151.64
	Q92841	DDX17	Probable ATP-dependent RNA helicase DDX17	9	5	7.27%	80272.66
	P11021	HSPA5	78 kDa glucose-regulated protein	6	4	8.41%	72332.86
	O14545	TRAFD1	TRAF-type zinc finger domain-containing protein 1	4	2	4.30%	64841.33
	Q9Y4W6	AFG3L2	AFG3-like protein 2	2	2	2.89%	88583.71
	Q13409	DYNC1L2	Cytoplasmic dynein 1 intermediate chain 2	3	1	3.29%	71456.69
	Q92945	KHSRP	Far upstream element-binding protein 2	1	1	1.97%	73115.45

	P35637	FUS	RNA-binding protein FUS	1	1	2.66%	53425.81
	Q92804	TAF15	TATA-binding protein-associated factor 2N	1	1	2.36%	61830.02
	Q8ND56	LSM14A	Protein LSM14 homolog A	1	1	2.38%	50530.01
	P52732	KIF11	Kinesin-like protein KIF11	1	1	0.85%	119158.9
	Q9P270	SLAIN2	SLAIN motif-containing protein 2	1	1	2.93%	62543.68
	P42166	TMPO	Lamina-associated polypeptide 2, isoform alpha	1	1	2.31%	75492.19
	Q00325	SLC25A3	Phosphate carrier protein, mitochondrial	1	1	7.18%	40094.92
SR7 (～70 kDa)	P11142	HSPA8	Heat shock cognate 71 kDa protein	56	17	26.78%	70897.96
	Q8WV44	TRIM41	E3 ubiquitin-protein ligase TRIM41	51	8	11.59%	71670.38
	P38646	HSPA9	Stress-70 protein, mitochondrial	11	8	13.40%	73680.41
	Q92841	DDX17	Probable ATP-dependent RNA helicase DDX17	12	6	10.84%	80272.66
	P34931	HSPA1L	Heat shock 70 kDa protein 1-like	8	5	10.45%	70374.95
	P08107	HSPA1A	Heat shock 70 kDa protein 1A/1B	8	5	9.67%	70052.18
	P17066	HSPA6	Heat shock 70 kDa protein 6	8	4	8.24%	71028.19
	P52272	HNRNPM	Heterogeneous nuclear ribonucleoprotein M	6	4	5.62%	77515.48
	Q9UNF1	MAGED2	Melanoma-associated antigen D2	5	3	5.45%	64954.31
	O14744	PRMT5	Protein arginine N-methyltransferase 5	5	3	5.02%	72684.05
	P40939	HADHA	Trifunctional enzyme subunit alpha, mitochondrial	4	3	4.59%	82999.58
	P35637	FUS	RNA-binding protein FUS	2	2	3.04%	53425.81
	Q9UJV9	DDX41	Probable ATP-dependent RNA helicase DDX41	2	1	1.77%	69837.79
	O75143	ATG13	Autophagy-related protein 13	1	1	2.13%	56571.92
	Q15054	POLD3	DNA polymerase delta subunit 3	1	1	2.58%	51400.24
	Q8WV28	BLNK	B-cell linker protein	1	1	1.97%	50466.54
	Q96AE4	FUBP1	Far upstream element-binding protein 1	1	1	2.02%	67560.45
	O95573	ACSL3	Long-chain-fatty-acid--CoA ligase 3	1	1	2.08%	80420.06

	Q9UG63	ABCF2	ATP-binding cassette sub-family F member 2	1	1	1.77%	71290.27
SR8 (～60-65 kDa)	Q9NVI7	ATAD3A	ATPase family AAA domain-containing protein 3A	9	6	9.94%	71369.32
	Q8WV44	TRIM41	E3 ubiquitin-protein ligase TRIM41	9	5	8.25%	71670.38
	O95831	AIFM1	Apoptosis-inducing factor 1, mitochondrial	7	4	7.83%	66900.63
	P49368	CCT3	T-complex protein 1 subunit gamma	7	4	8.07%	60533.83
	P28288	ABCD3	ATP-binding cassette sub-family D member 3	2	2	4.40%	75475.99
	E5RGY8	OSR2	Protein odd-skipped-related 2 (Fragment)	1	1	3.70%	30107.96
	P17844	DDX5	Probable ATP-dependent RNA helicase DDX5	1	1	1.79%	69148.13
	Q96CJ1	EAF2	ELL-associated factor 2	1	1	4.23%	28791.63
	Q9UFD9	RIMBP3	RIMS-binding protein 3A	1	1	0.92%	180718.37
	P61978	HNRNPK	Heterogeneous nuclear ribonucleoprotein K	1	1	2.38%	50976.43
SR9 (～55 kDa)	P07437	TUBB	Tubulin beta chain	142	20	56.31%	49670.85
	P68371	TUBB4B	Tubulin beta-4B chain	123	20	56.18%	49831.04
	P04350	TUBB4A	Tubulin beta-4A chain	117	18	49.55%	49585.81
	Q13885	TUBB2A	Tubulin beta-2A chain	116	15	38.20%	49907.02
	Q13509	TUBB3	Tubulin beta-3 chain	106	11	27.56%	50432.75
	Q9BUF5	TUBB6	Tubulin beta-6 chain	66	6	14.35%	49857.16
	P31943	HNRNPH 1	Heterogeneous nuclear ribonucleoprotein H	17	4	12.25%	49229.59
	P25705	ATP5A1	ATP synthase subunit alpha, mitochondrial	6	4	10.31%	59750.67
	Q9Y230	RUVBL2	RuvB-like 2	5	3	7.78%	51156.58
	Q71U36	TUBA1A	Tubulin alpha-1A chain	4	3	10.86%	50135.7
	P68363	TUBA1B	Tubulin alpha-1B chain	4	3	10.86%	50151.71
	Q9BQE3	TUBA1C	Tubulin alpha-1C chain	4	3	10.91%	49895.39

	Q13748	TUBA3C	Tubulin alpha-3C/D	4	3	10.89%	49959.62
	Q1ZYQ1	TUBA2	Tubulin, alpha 2	4	3	10.89%	49959.62
	Q8WV44	TRIM41	E3 ubiquitin-protein ligase TRIM41	4	3	5.24%	71670.38
	Q15084	PDIA6	Protein disulfide-isomerase A6	3	2	4.09%	48121.38
	P36578	RPL4	60S ribosomal protein L4	2	1	2.58%	47697.19
	Q8NEY1	NAV1	Neuron navigator 1	2	1	0.64%	202472.16
	P56182	RRP1	Ribosomal RNA processing protein 1 homolog A	2	1	2.60%	52839.4
	P08670	VIM	Vimentin	2	1	3.00%	53651.88
	Q68CQ1	HEATR8	HEAT repeat-containing protein 8	1	1	0.83%	145647.13
	B7ZBM2	C1orf175	Chromosome 1 open reading frame 175	1	1	1.23%	100361.73
	P68104	EEF1A1	Elongation factor 1-alpha 1	1	1	2.38%	50140.69
	P19474	TRIM21	E3 ubiquitin-protein ligase TRIM21	1	1	1.89%	54169.86
	P39656	DDOST	Dolichyl-diphosphooligosaccharide--protein glycosyltransferase 48 kDa subunit	1	1	2.41%	50800.96
	Q13625	TP53BP2	Apoptosis-stimulating of p53 protein 2	1	1	1.06%	125616.74
	Q8NDQ6	ZNF540	Zinc finger protein 540	1	1	2.58%	77093.81
	Q8TAG5	VSTM2A	V-set and transmembrane domain-containing protein 2A	1	1	6.36%	25833.34
	Q14257	RCN2	Reticulocalbin-2	1	1	3.79%	36876.63
SR10 (～43-50 kDa)	P49411	TUFM	Elongation factor Tu, mitochondrial	5	3	8.41%	49541.51
	P62736	ACTA2	Actin, aortic smooth muscle	4	3	9.81%	42009.01
	P60709	ACTB	Actin, cytoplasmic 1	4	3	9.87%	41736.77
	P68032	ACTC1	Actin, alpha cardiac muscle 1	4	3	9.81%	42019.01
	P63261	ACTG1	Actin, cytoplasmic 2	4	3	9.87%	41792.89
	P63267	ACTG2	Actin, gamma-enteric smooth muscle	4	3	9.84%	41876.93
	P68133	ACTA1	Actin, alpha skeletal muscle	4	3	9.81%	42051.06

	Q14103	HNRNPD	Heterogeneous nuclear ribonucleoprotein D0	2	1	3.94%	38434.13
	O60884	DNAJA2	DnaJ homolog subfamily A member 2	2	1	3.16%	45745.65
	Q9BQA1	WDR77	Methylosome protein 50	2	1	4.39%	36724.46
	P31689	DNAJA1	DnaJ homolog subfamily A member 1	2	1	3.27%	44868.19
	P50454	SERPINH 1	Serpin H1	1	1	2.63%	46440.56
	Q12872	SFSWAP	Splicing factor, suppressor of white-apricot homolog	1	1	1.05%	104822.06
	Q4U2R8	SCL22A6	Solute carrier family 22 member 6	1	1	2.13%	61816.37
	O75821	EIF3G	Eukaryotic translation initiation factor 3 subunit G	1	1	2.50%	35611.01
SR11 (~35-40 kDa)	P54105	CLNS1A	Methylosome subunit pICln	3	1	5.49%	26215.48
	O75477	ERLIN1	Erlin-1	2	1	3.47%	38925.84
	Q02878	RPL6	60S ribosomal protein L6	1	1	3.12%	32727.79
	P05109	S100A8	Protein S100-A8	1	1	11.83%	10834.49
	Q16637	SMN1	Survival motor neuron protein	1	1	3.40%	31848.78
	Q9P035	PTPLAD1	Very-long-chain (3R)-3-hydroxyacyl-[acyl-carrier protein] dehydratase 3	1	1	2.49%	43159.62
	P61626	LYZ	Lysozyme C	1	1	6.08%	16536.97
	Q9Y3F4	STRAP	Serine-threonine kinase receptor-associated protein	1	1	3.43%	38438.33
	Q01081	U2AF1	Splicing factor U2AF 35 kDa subunit	1	1	5.42%	27872.19
	Q86YZ3	HRNR	Hornerin	1	1	0.35%	282390.98
	Q9BWF3	RBM4	RNA-binding protein 4	1	1	4.40%	40313.98
SR12 (~30 kDa)	P12004	PCNA	Proliferating cell nuclear antigen	6	3	14.94%	28768.8
	P50402	EMD	Emerin	5	3	14.57%	28994.06
	Q02878	RPL6	60S ribosomal protein L6	3	2	5.21%	32727.79

	P22087	FBL	rRNA 2'-O-methyltransferase fibrillarin	3	2	6.85%	33784.17
	P68104	EEF1A1	Elongation factor 1-alpha 1	2	1	2.38%	50140.69
	Q05639	EEF1A2	Elongation factor 1-alpha 2	2	1	2.38%	50470.03
	Q5VTE0	EEF1A1P5	Putative elongation factor 1-alpha-like 3	2	1	2.38%	50184.85
	P05109	S100A8	Protein S100-A8	2	1	11.83%	10834.49
	P04406	GAPDH	Glyceraldehyde-3-phosphate dehydrogenase	2	1	4.48%	36053.05
	O00165	HAX1	HCLS1-associated protein X-1	2	1	3.94%	31620.76
	Q9UEU0	VTI1B	Vesicle transport through interaction with t-SNAREs homolog 1B	2	1	4.74%	26688.49
	Q8NHW5	RPLP0P6	60S acidic ribosomal protein P0-like	1	1	3.79%	34364.47
	P43307	SSR1	Translocon-associated protein subunit alpha	1	1	3.85%	32235.45
	O15460	P4HA2	Prolyl 4-hydroxylase subunit alpha-2	1	1	1.68%	60902.07
	P62805	HIST1H4A	Histone H4	1	1	9.71%	11367.28
	P61964	WDR5	WD repeat-containing protein 5	1	1	3.29%	36588.36
	Q9H4D0	CLSTN2	Calsyntenin-2	1	1	1.15%	107005.88
	P14866	HNRNPL	Heterogeneous nuclear ribonucleoprotein L	1	1	3.06%	64132.9
	Q6P4A7	SFXN4	Sideroflexin-4	1	1	4.45%	37998.44
	Q86YZ3	HRNR	Hornerin	1	1	0.35%	282390.98
SR13 (≤25 kDa)	P05141	SLC25A5	ADP/ATP translocase 2	11	6	19.46%	32852.17
	P12236	SLC25A6	ADP/ATP translocase 3	10	5	17.79%	32866.21
	P62701	RPS4X	40S ribosomal protein S4, X isoform	6	3	11.41%	29597.64
	P25789	PSMA4	Proteasome subunit alpha type-4	4	3	14.56%	29483.81
	P23396	RPS3	40S ribosomal protein S3	4	3	16.87%	26688.31

P27348	YWHAQ	14-3-3 protein theta	3	2	8.98%	27764.29
Q9Y657	SPIN1	Spindlin-1	2	2	8.78%	29600.55
Q02978	SLC25A1 1	Mitochondrial 2-oxoglutarate/malate carrier protein	2	1	5.10%	34061.66
P53007	SLC25A1	Tricarboxylate transport protein, mitochondrial	1	1	3.54%	34012.64
Q9NPF8	ADAP2	Arf-GAP with dual PH domain-containing protein 2	1	1	2.89%	44348.97
Q9H5I5	PIEZO2	Piezo-type mechanosensitive ion channel component 2	1	1	0.36%	318064.72
Q5T653	MRPL2	39S ribosomal protein L2, mitochondrial	1	1	3.28%	33300.77
P62917	RPL8	60S ribosomal protein L8	1	1	4.28%	28024.58
Q6PQ33	LINC0027 3	Protein LINC00273	1	1	1.52%	49322.27
P18124	RPL7	60S ribosomal protein L7	1	1	4.44%	29225.74
Q00325	SLC25A3	Phosphate carrier protein, mitochondrial	1	1	3.31%	40094.92
Q9UEU0	VTI1B	Vesicle transport through interaction with t-SNAREs homolog 1B	1	1	4.74%	26688.49
Q9NPL8SV=2	TIMMDC 1	Translocase of inner mitochondrial membrane domain-containing protein 1	1	1	3.86%	32177.82
Q9Y5M8	SRPRB	Signal recognition particle receptor subunit beta	1	1	7.01%	29702.23

[a] The TRIM41-associated proteins in TRIM41-Flag-transfected HEK293T cells after immunoprecipitation were analyzed by LC-MS. SR (subregion) 1-13 represent different regions in the gel with different molecular weight.

[b] Note that the house-keeping proteins, such as kinesins, actins, tubulins, heat shock proteins and translation factors, have not been excluded from the lists.

[c] The fonts of TRIM41 and the TRIM41-aasociated proteins that have been reported to be involved in inflammatory or infectious processes are in red.

Table S2. Sequences used in this study.

**Primers for genotyping of *Trim41* knockout mice**		
Primer name	Primer sequence (5'→3')	Result judgment
P1	CTCCATGGTTTGTTCAGTTGGAG	P1+P2 (~600 bp)<br>Positive: −/− or +/−<br>Negative: +/+
P2	CTTGGCTGCAGGTCGTCGAAATT C	
P3	GGAACCACTTAGGAAGCATCTAG	P3+P4 (~750 bp)<br>Positive: +/+ or +/−<br>Negative: −/− or +/−
P4	CAATCACAATGTGCTATCTGGC	
***Bcl10*-specific targeted sequences for CRISPR-Cas9 edition**		
Sequence No.	Sequence	KO efficiency
1	GCCGAACTTCAAGTAGAAAA	No
2	GATGAGGTGCTAAAGCTT	No
3	GCCACCAACAACCTCTCT	Yes
4	CGGCTCCCTTCTTCTCTA	Yes
***Nemo*-specific targeted sequences for CRISPR-Cas9 edition**		
Sequence No.	Sequence	KO efficiency
1	GCATGTCCTGGTCCTCTGC	Yes
2	AGCAGAGGACCAGGACATGC	No
3	AGGATCGGCAAGCTTTAG	No
4	GCAAGGCAGCCTCCACGCTC	No
***Trim41*-specific siRNA sequences**		
Sequence name	Sequence (5'→3')	Knockdown efficiency
siRNA1	CCAUCUGCCUCGAUUACUUTT	Highest and selected
	AAGUAAUCGAGGCAGAUGGTT	
siRNA2	GGAACCACUUAGGAAGCAUTT	High
	AUGCUUCCUAAGUGGUUCCTT	

siRNA3	GCGCCUAGGCUUCUACAAUTT	High
	AUUGUAGAAGCCUAGGCGCTT	
**Primers for Q-PCR assays**		
Gene name	Primer sequence (5’→3’)	
	Sense	Anti-sense
Il6	GAGTTGTGCAATGGCAATTCTG	GCAAGTGCATCATCGTTGTTCA T
Ifnb	CAGCTCCAAGAAAGGACGAAC	GGCAGTGTAACTCTTCTGCAT
Tnf	CCCTCACACTCAGATCATCTTCT	GCTACGACGTGGGCTACAG
Actb	AGTGTGACGTTGACATCCGT	GCAGCTCAGTAACAGTCCGC

Table S3. Antibodies used in this study.

Company	Product name	Clone No.	Cat. No.
Cell Signaling Technology (Beverly, MA)	Anti- IKKα/β (Ser176/Ser180)	C84E11	2078
	Anti- IKKβ	D30C6	8943
	Anti- IκBα	44D4	4812
	Anti- IκBα (Ser32/Ser36)	5A5	9246
	Anti-BCL10	C78F1	4237
	Anti-CARD11	1D12	4435
	Anti-CARD9	NA	12283
	Anti-ERK1/2	137F5	4695
	Anti-ERK1/2 (Thr202/Tyr204)	D13.14.4E	4370
	Anti-Flag tag	9A3	8146
	Anti-GAPDH	D16H11	5174
	Anti-HA tag	C29F4	3724
	Anti-IRF3	D83B9	4302
	Anti-JNK1/2	56G8	9258
	Anti-JNK1/2 (Thr183/Tyr185)	81E11	4668
	Anti-MAVS	NA	4983
	Anti-Myc tag	71D10	2278
	Anti-p38	D13E1	8690
	Anti-p38 (Thr180/Tyr182)	12F8	4631
	Anti-p65	L8F6	6956
	Anti-phospho-IRF3 (Ser396)	4D4G	4947
	Anti-phospho-TBK1 (Ser172)	D52C2	5483
	Anti-STING	D2P2F	13647
	Anti-TBK1	D1B4	3504
	Anti-Ubiquitin	P4D1	3936
Bethyl Labs	Anti-ADAR1	NA	A303-884A

Abcam Inc. (Cambridge, MA)	Anti-HSV-tag	NA	ab19354
	Anti-Lamin A	133A2	ab8980
	Anti-TREX1	NA	ab83890
	Anti-TRIM41	NA	ab98170
MBL International	Anti-IKK gamma	EA2-6	K0159-3

NA, not applicable.
