# Supplementary material for: TRIM41 is required to innate antiviral response by polyubiquitinating BCL10 and recruiting NEMO
Source: Signal Transduct Target Ther. 2021 Feb 28;6:90. doi: 10.1038/s41392-021-00477-8 (PMC7914255; doi:10.1038/s41392-021-00477-8)

Figure 1e

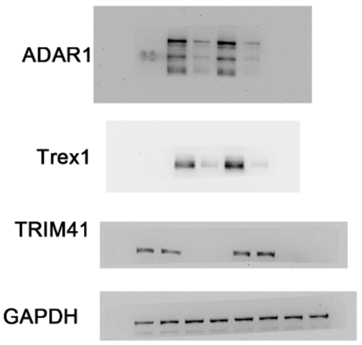

Fig S3

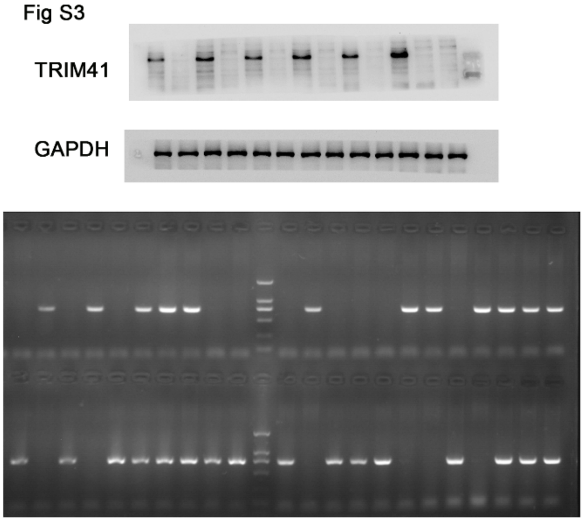

Genotyping of
Trim41 mice

Figure S1b

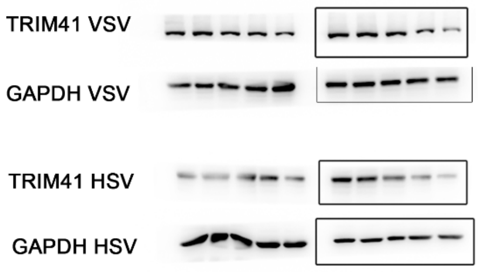

Fig 2f

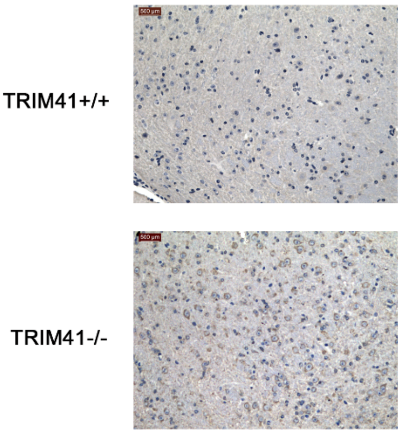

BRAIN

Figure S1c

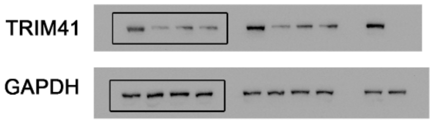

Figure S2e

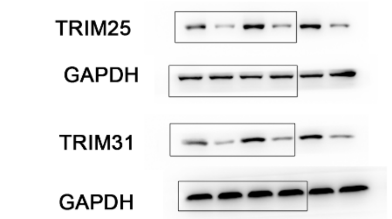

Figure S2f

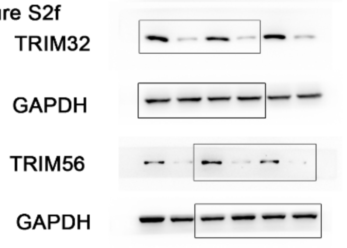

Fig 3a

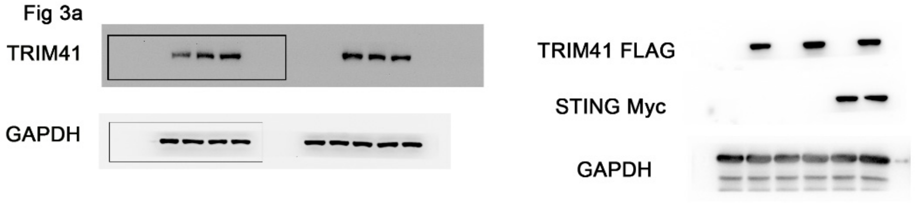
TRIM41
GAPDH
TRIM41 FLAG
STING Myc
GAPDH

Fig 3f

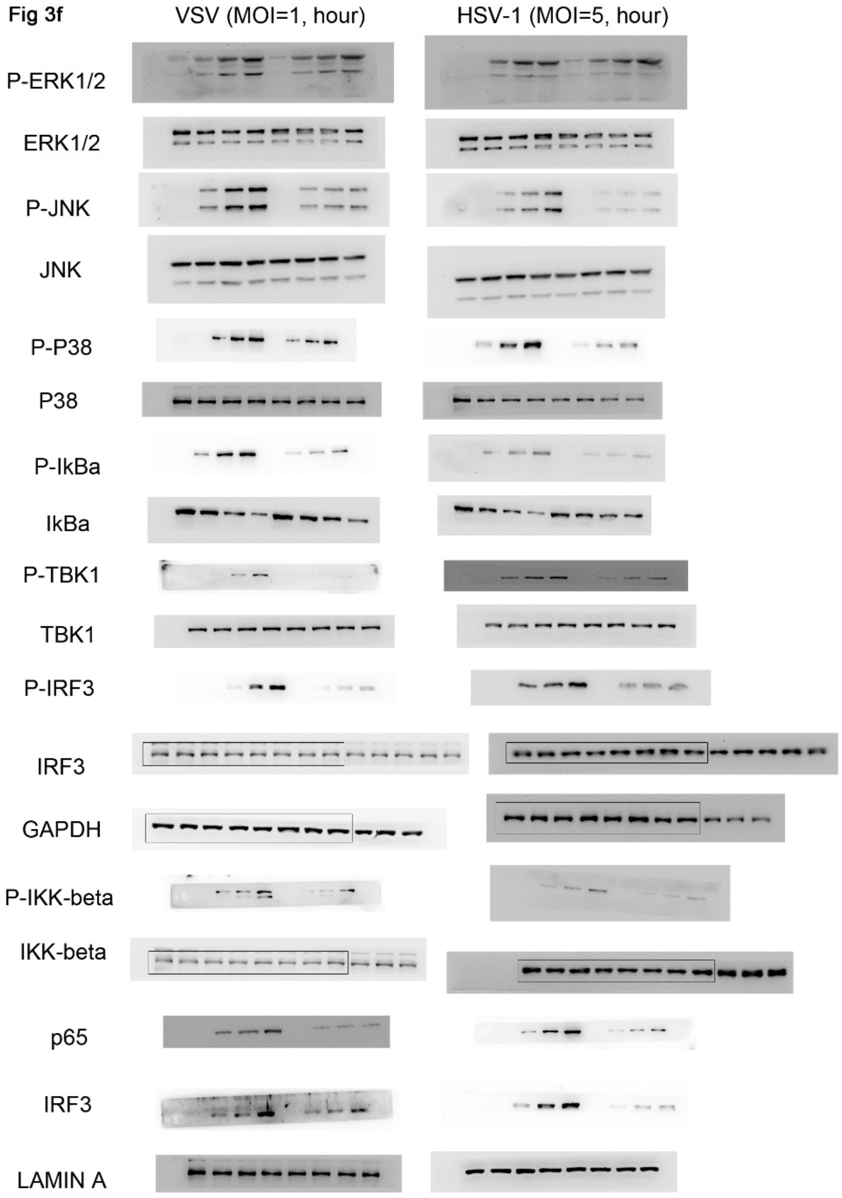
VSV (MOI=1, hour)
HSV-1 (MOI=5, hour)
P-ERK1/2
ERK1/2
P-JNK
JNK
P-P38
P38
P-IkBa
IkBa
P-TBK1
TBK1
P-IRF3
IRF3
GAPDH
P-IKK-beta
IKK-beta
p65
IRF3
LAMIN A

Fig 4a

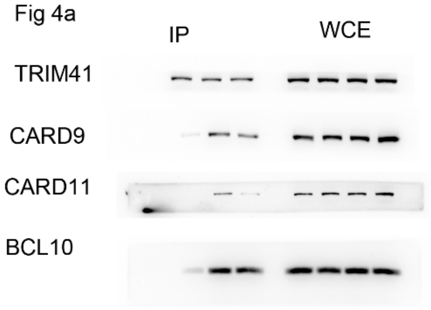

Fig 4b

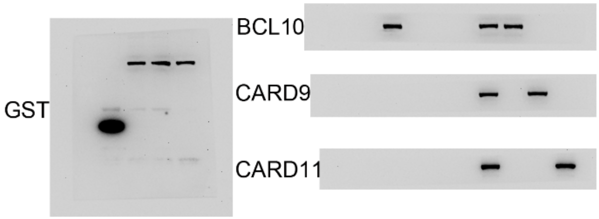

Fig 4d

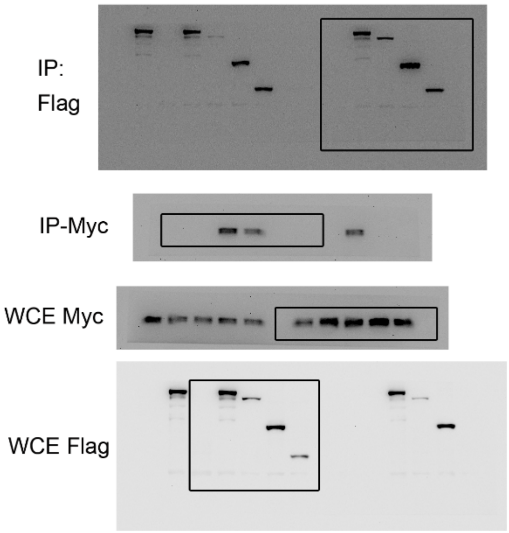

Fig 4e

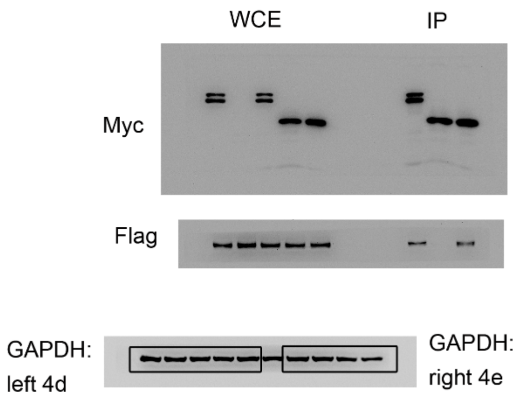

Fig 4f

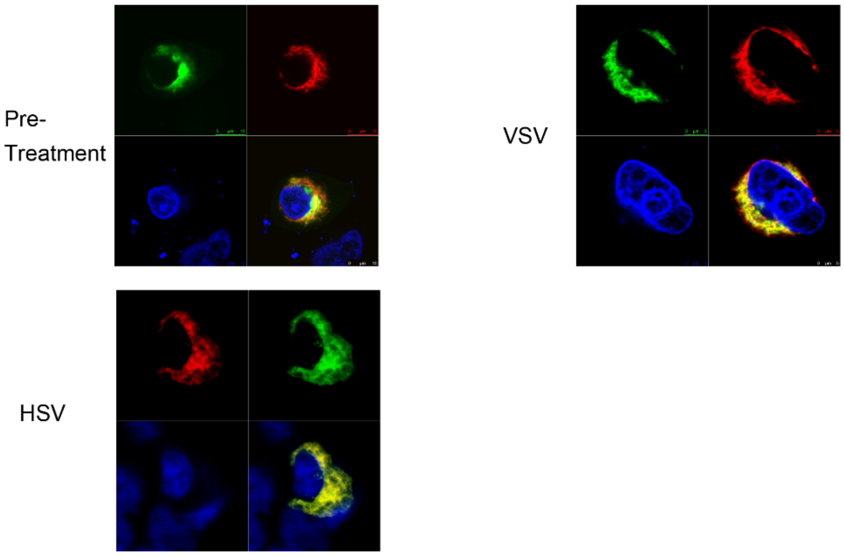

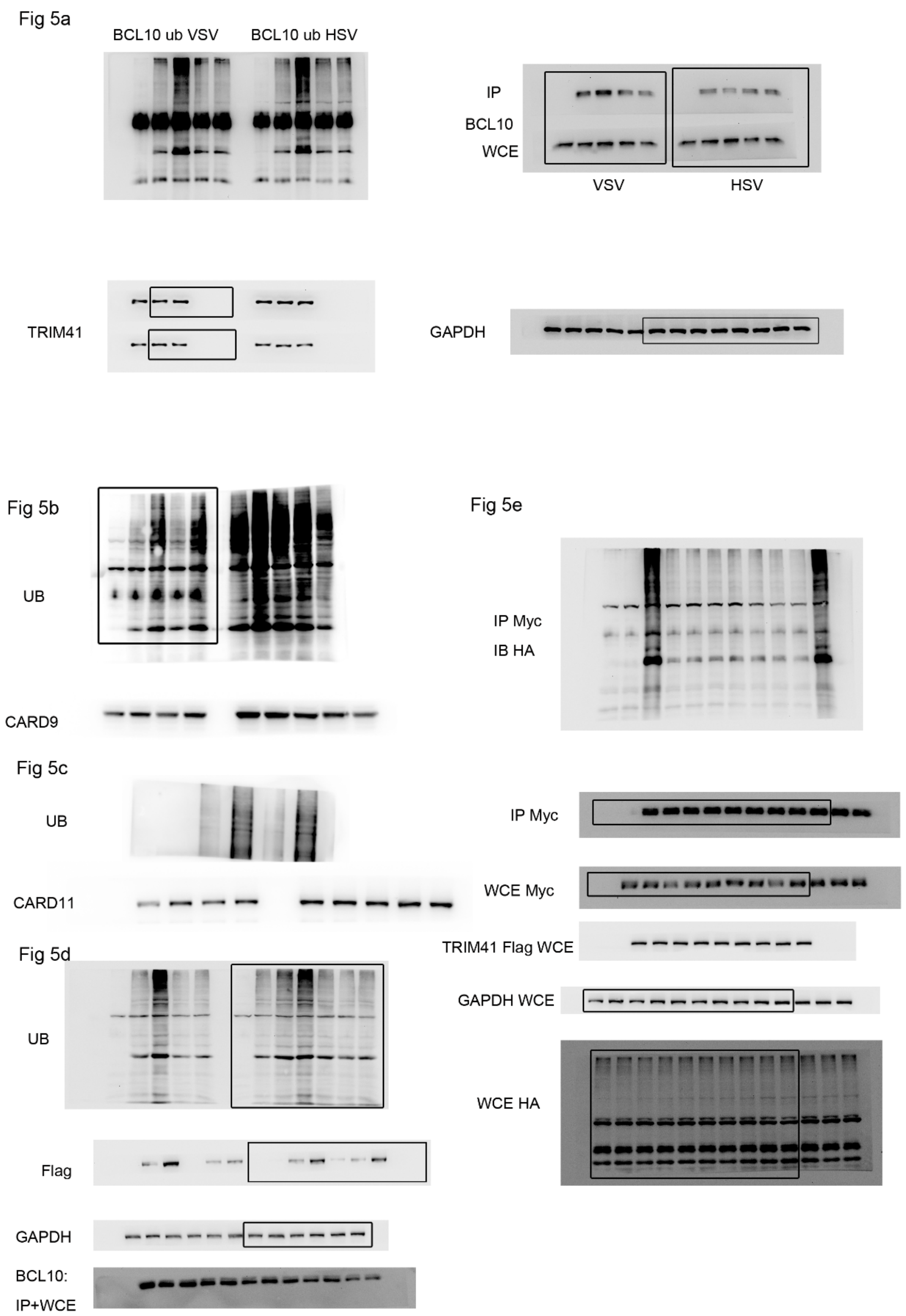

Fig 5a
BCL10 ub VSV
BCL10 ub HSV
IP
BCL10
WCE
VSV
HSV
TRIM41
GAPDH
Fig 5b
UB
CARD9
Fig 5e
IP Myc
IB HA
Fig 5c
UB
CARD11
IP Myc
WCE Myc
TRIM41 Flag WCE
GAPDH WCE
Fig 5d
UB
WCE HA
Flag
GAPDH
BCL10:
IP+WCE

Fig 5f

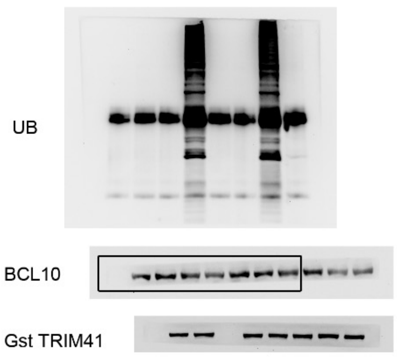

Fig 5g

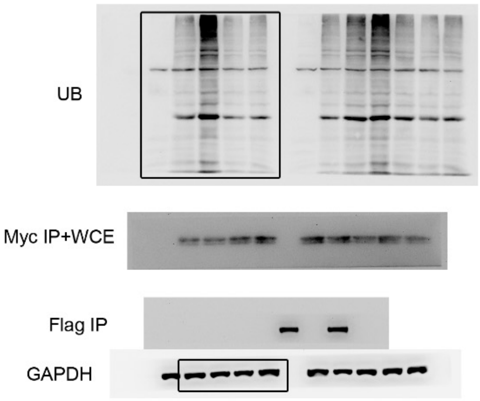

Fig 6a-6b

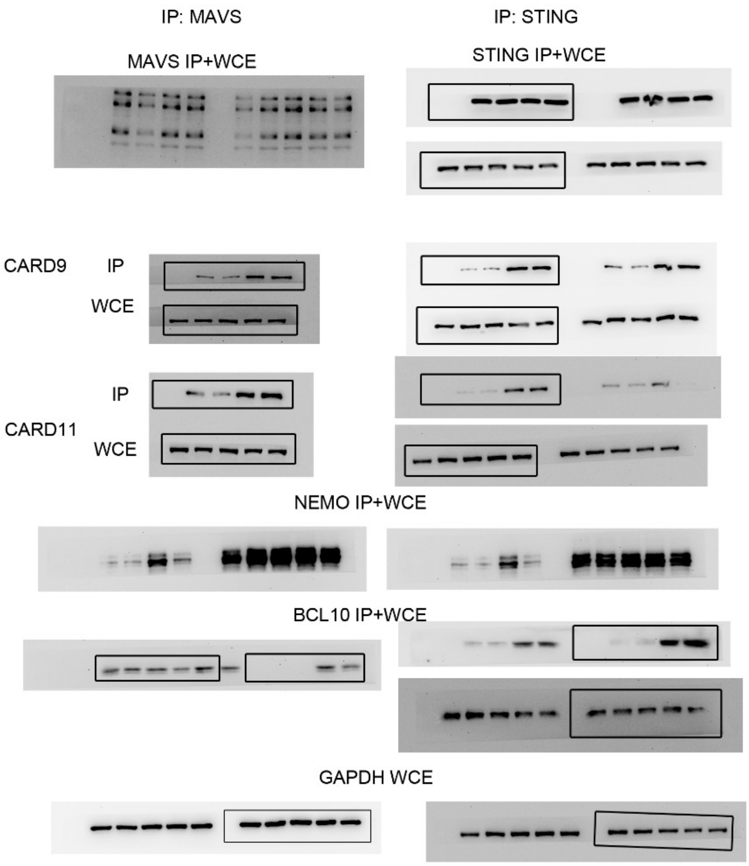

Fig S5a

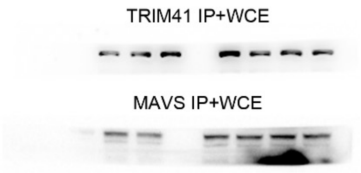

Fig S5b

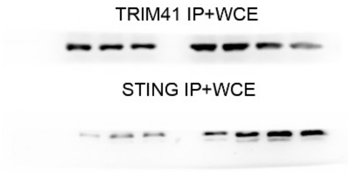

Fig 6c

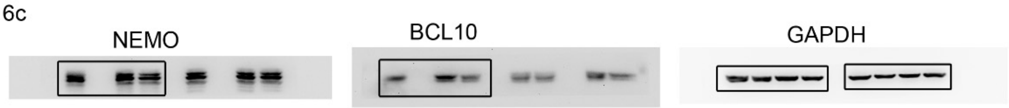

Fig 6e-6f

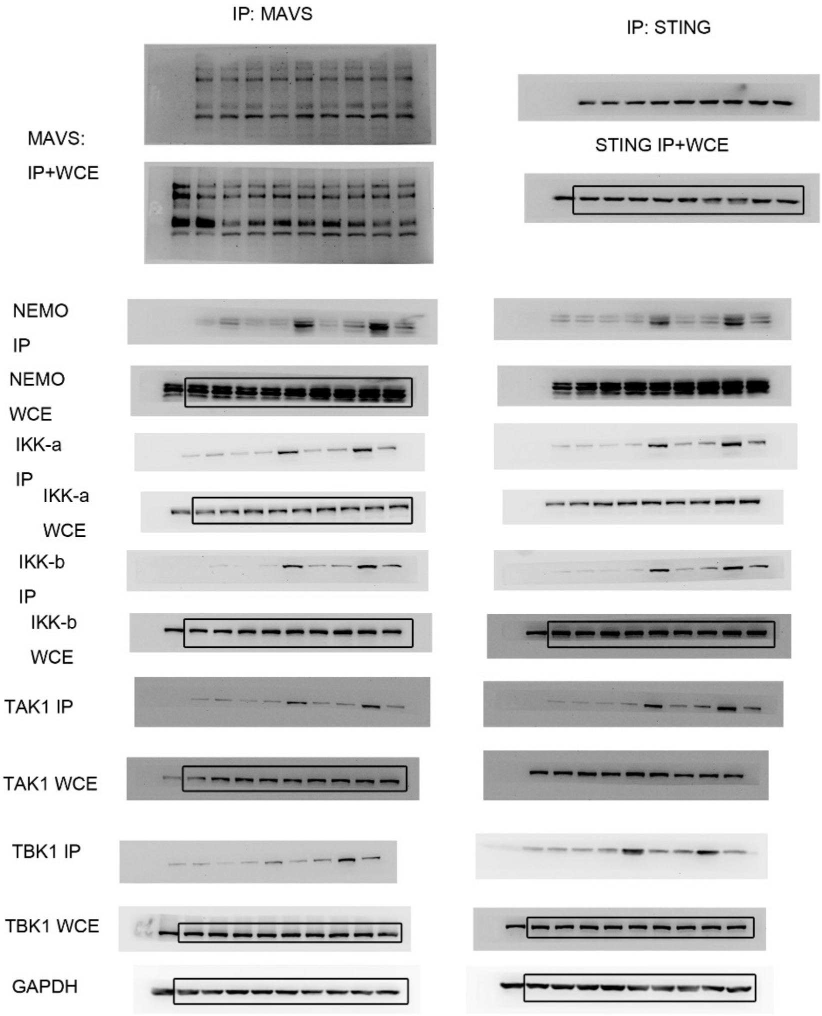

Supplement: Supplementary file 2 — Dataset 1 [file 41392_2021_477_MOESM2_ESM.pdf]
